# Macrophage PPAR gamma Co-activator-1 alpha participates in repressing foam cell formation and atherosclerosis in response to conjugated linoleic acid

**DOI:** 10.1002/emmm.201302587

**Published:** 2013-08-21

**Authors:** Cathal McCarthy, Nora T Lieggi, Denis Barry, Declan Mooney, Monica de Gaetano, William G James, Sarah McClelland, Mary C Barry, Laure Escoubet-Lozach, Andrew C Li, Christopher K Glass, Desmond J Fitzgerald, Orina Belton

**Affiliations:** 1 School of Biomolecular and Biomedical Science, UCD Conway Institute, UCDDublin, Ireland; 2 Department of Vascular Surgery, St. Vincent's University HospitalDublin, Ireland; 3 Department of Cellular and Molecular Medicine, UCSDCA, USA; 4 Department of Medicine, UCSDCA, USA; 5 School of Medicine and Medical Sciences, UCD Conway Institute, UCDDublin, Ireland

**Keywords:** atherosclerosis regression, conjugated linoleic acid, foam cell formation, PGC-1α

## Abstract

Conjugated linoleic acid (CLA) has the unique property of inducing regression of pre-established murine atherosclerosis. Understanding the mechanism(s) involved may help identify endogenous pathways that reverse human atherosclerosis. Here, we provide evidence that CLA inhibits foam cell formation via regulation of the nuclear receptor coactivator, peroxisome proliferator-activated receptor (PPAR)-γ coactivator (PGC)-1α, and that macrophage PGC-1α plays a role in atheroprotection *in vivo*. PGC-1α was identified as a hub gene within a cluster in the aorta of the apoE^−/−^ mouse in the CLA-induced regression model. PGC-1α was localized to macrophage/foam cells in the murine aorta where its expression was increased during CLA-induced regression. PGC-1α expression was also detected in macrophages in human atherosclerosis and was inversely linked to disease progression in patients with the disease. Deletion of PGC-1α in bone marrow derived macrophages promoted, whilst over expression of the gene inhibited foam cell formation. Importantly, macrophage specific deletion of PGC-1α accelerated atherosclerosis in the LDLR^−/−^ mouse *in vivo*. These novel data support a functional role for PGC-1α in atheroprotection.

## INTRODUCTION

Conjugated linoleic acid (CLA) is a generic term denoting a group of naturally occurring isomers of linoleic acid (18:2, n6), which vary in the position and geometry of their double bonds (Mitchell & McLeod, 2008). CLA has anti-atherogenic properties, inhibiting the progression of atherosclerosis in animal models (Kritchevsky et al, 2004; Lee et al, 1994). We have previously shown that dietary administration of a 1% CLA blend (80:20, *cis*9,*trans*11-CLA:*trans*10,*cis*12-CLA) induces regression of pre-established atherosclerosis in the apoE^−/−^ mouse model despite continuing a high cholesterol challenge (Toomey et al, 2006). Understanding the mechanisms involved through which CLA mediates regression may help identify endogenous pathways that limit or reverse human atherosclerosis.

Previously, we reported that macrophage cell accumulation was reduced in the atherosclerotic plaques of CLA-fed mice suggesting this cell as a potential target for CLA (Toomey et al, 2006). Other studies have shown that CLA isomers reduce cholesterol accumulation in macrophage-derived foam cells potentially by enhancing lipid acceptor-dependent cholesterol efflux (Ringseis et al, 2008). Thus, altered cholesterol handling by macrophages may in part explain how CLA mediates its atheroprotective effect. However, other evidence suggests that CLA modulates the macrophage pro-inflammatory phenotype, with suppression of pro-inflammatory genes such as VCAM-1, matrix metalloproteinase (MMP)-9, platelet endothelial cell adhesion molecule (PECAM)-1 and pro-inflammatory cytokines interleukin (IL)-1α, IL-1β and IL-6 (Lee et al, 2009; Toomey et al, 2006). We have also shown that CLA inhibits the inflammatory phenotype of the monocyte/macrophage with reduced COX-2 and cPLA_2_ expression and inhibition of PGE_2_ and MMP-9 generation (McClelland et al, 2010). The findings cumulatively point to the monocyte/macrophage as an important cellular target for CLA in atherosclerosis.

One of the proposed mechanisms for the effects of CLA is activation of peroxisome proliferator-activated receptors (PPARs). Activated PPARs heterodimerize with the retinoic X receptor (RXR) and bind to specific peroxisome proliferator response elements (PPREs) located in the promoter of target genes thus regulating their transcription. Activators of PPARα and PPARγ inhibit foam cell formation (Li et al, 2004). Inhibition of foam-cell formation by PPARα is dependent on regulation of the nuclear receptor, liver X receptor α (LXRα), which regulates genes involved in cholesterol homeostasis and lipid metabolism, while activation of PPARγ stimulates HDL-dependent cholesterol efflux and inhibition of foam cell formation independently of LXRα (Li et al, 2004). *In vivo*, PPARγ agonists inhibit the development of atherosclerosis in the LDLR^−/−^ and apoE^−/−^ murine models (Chen et al, 2001; Li et al, 2000). Importantly, while activation of PPARγ inhibits the development of atherosclerosis, regression of atherosclerosis is not induced (Nakaya et al, 2009), which is in contrast to the regression seen with CLA supplementation (Toomey et al, 2004). Thus, PPAR activation alone cannot explain the response to CLA. Indeed, PPAR agonists and CLA differ in their effects on monocytes. We recently reported that both CLA and the PPARγ agonist, troglitazone, inhibit MCP-1-induced monocyte migration. However, only CLA inhibited platelet-releasate-induced migration implying a PPARγ-independent mechanism for CLA-induced suppression of monocyte migration (McClelland et al, 2010).

In this study we employed a comprehensive transcriptomic approach to identify novel gene clusters regulated by CLA during atherosclerosis regression *in vivo*. The implications of this are important as to date there is limited knowledge of gene networks which explain how atherosclerosis could be reversed *in vivo*. In a screen of genes regulated during CLA-mediated regression of atherosclerosis, we identified several gene clusters, one of which contained the gene PGC-1α at its hub. Given the role of PGC-1α in regulating genes involved in lipid metabolism and its emerging role in vascular cell function we pursued PGC-1α as a potential target of CLA induced atherosclerosis regression.

PGC-1α is a transcriptional coactivator of several nuclear receptors that regulate key metabolic steps in energy homeostasis (Puigserver & Spiegelman 2003), including PPARγ. PGC-1α is expressed in tissues such as heart and skeletal muscle where it regulates mitochondrial biogenesis via the regulation of genes involved in fatty acid oxidation and oxidative phosphorylation. PGC1-α also plays a role in vascular biology. PGC-1α is expressed in VSMCs and endothelial cells (Kim et al, 2007). Overexpression of PGC-1α blocks oleic acid (OA)-induced VSMC proliferation and migration (Zhang et al, 2007) and PGC-1α has been shown to mediate the inhibitory effect of dexamethasone on PDGF-induced VSMC migration (Xu et al, 2010). PGC-1α also inhibits neointimal formation in the rat carotid artery (Qu et al, 2009). In endothelial cells, PGC-1α triggered an increase in the expression of mitochondrial antioxidative enzymes (Valle et al, 2005), and prevented linoleic acid-induced increases in ROS generation and apoptosis by increasing fatty acid oxidation. In addition, PGC-1α serves as a coactivator of LXRα (Oberkofler et al, 2003) and activates Cyp7A1 expression (Shin et al, 2003), a crucial enzyme in mammalian cholesterol metabolism in the liver, where it mediates the classical pathway of bile acid synthesis (Chiang, 2002). These findings point to PGC-1α as a potential therapeutic target in atherosclerosis (Won et al, 2010).

Here, we identify regulation of a PGC-1α network during regression of pre-established atherosclerosis. We further extended our studies to explore the expression of PGC-1α in murine models and human atherosclerosis. Importantly we describe the effect of CLA and PGC-1α on macrophage to foam cell transition. Individual CLA isomers as well as the 80:20 CLA blend, which is atheroprotective *in vivo* was examined for their effect on oxLDL uptake and macrophage foam cell formation and on the expression of PGC-1α⋅ Finally, we directly explored the role of PGC-1α in regulating foam cell formation, over-expressing the gene *in vitro* and using PGC-1α depleted macrophages *in vivo*.

Our data shows for the first time that CLA regulates PGC-1α expression in macrophages *in vitro* and *in vivo* in murine and human atherosclerosis. Furthermore, we show that PGC-1α expression inhibits macrophage- foam cell transition. Finally for the first time we provide evidence, that macrophage specific PGC-1α depletion accelerates atherosclerosis *in vivo*, strongly supporting a role for PGC-1α in atheroprotection.

## RESULTS

### PGC-1α expression is increased in CLA-induced regression of atherosclerosis in the apoE^−/−^ model

**Figure 1:**
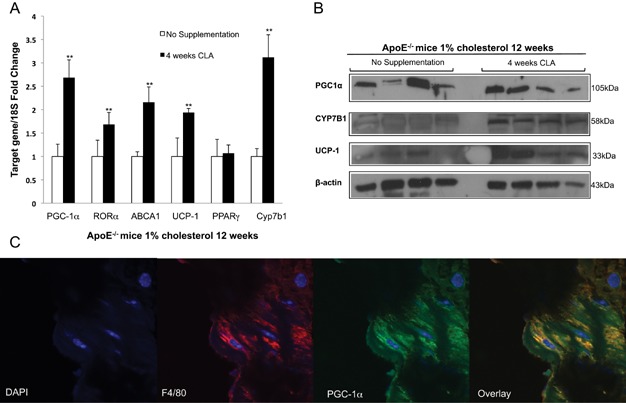
PGC-1α expression in apoE^−/−^ mouse PGC1α was identified as a central hub gene located in the network most represented by genes significantly increased with CLA supplementation *in vivo*. Validation of gene network *in vivo* showing mRNA expression of PGC-1α, RORα and ABCA1 and expression of known (PPARγ1 and UCP-1) and putative (CYP7b1) PGC-1α target genes in aorta of apoE^−/−^ fed a 1% cholesterol chow for 12 weeks with (*n* = 5) or without (*n* = 5) 1% CLA supplementation for the final 4 weeks.Western blot analysis of PGC-1α, UCP-1 and Cyp7b1 confirmed CLA-mediated induction of PGC-1α in murine aorta.PGC-1α (green) was localized to macrophages as confirmed by confocal microscopy staining with F4/80 (red) specific macrophage marker. Overlay shows co-localization of PGC-1α and F4/80. Nuclei are stained with DAPI and are show in blue. Data are the mean ± SEM of five independent experiments. Statistical analyses were performed using *t*-test. Significant *p*-values from left to right are as follows: *p* = 0.0013, 0.0103, 0.0054, 0.0092 and 0.0045 for PGC-1α, RORα, ABCA1, UCP-1 and Cyp7b1, respectively, versus apoE^−/−^ 1% cholesterol 12 weeks.

We have previously shown that 4 week supplementation of CLA blend induces regression of atherosclerosis in the apoE^−/−^ mouse (Toomey et al, 2006). Affymetrix gene arrays were performed on aortae from apoE^−/−^ mice, fed a 1% cholesterol chow for 12 weeks, supplemented with/without 1% CLA blend (80:20 *cis*-9,*trans*-11-CLA:*trans-*10,*cis*-12-CLA) for the final 4 weeks. Subsequent Ingenuity Pathway Analysis (IPA) was used to further elucidate the networks mapping CLA regulated genes to their appropriate pathways. Using stringent filtering of our data set to identify genes with a significant 1.7-fold differential expression, we identified PGC-1α as a hub gene within a network which was induced during CLA-mediated regression (Supporting Information Fig S1A). Transcriptomic data was further validated. Decreased lesion burden (Supporting Information Fig S1B) was associated with a significant increase in *Pgc-1α* gene expression (1 vs. 2.68 ± 0.72-fold, *p* = 0.0013), and expression of other genes identified on the network including *Abca1* (1 vs. 2.15 ± 0.33-fold, *p* = 0.0054) and *Rorα* (1 vs. 1.68 ± 0.26-fold, *p* = 0.0103) in the aorta of CLA fed animals, confirming regulation of the PGC-1α network in CLA-induced regression (Fig 1A). We subsequently examined regulation of known PGC-1α target genes. Although there was no significant change in the expression of PPARγ between study groups, there was increased expression of *Ucp1*, a known PGC-1α target gene (Puigserver et al, 1998) and *Cyp7b1*, in the aortae of CLA supplemented mice (Fig 1A and B). We localized the increase in PGC-1α protein expression in the CLA fed animals to the macrophage cell by immunohistochemical analysis using the F4/80 macrophage specific marker (Supporting Information Fig S2) and by confocal microscopy (Fig 1C). Furthermore, we scanned regions of co-localization (60×, oil) in an optimized 3D z-stack of 16 sequential slices, and performed analysis using the IMARIS software package (Supporting Information Movie S1). Together these data indicate that PGC-1α is regulated by CLA during regression of atherosclerosis and is expressed in macrophage cells *in vivo*.

### PGC-1α is differentially expressed in human symptomatic and asymptomatic atherosclerotic plaques

**Figure 2:**
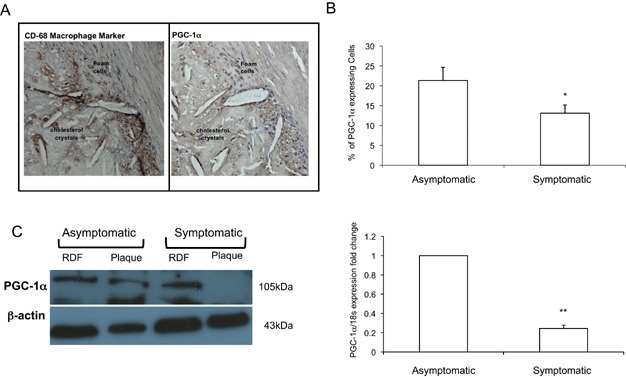
PGC-1α expression in human atherosclerosis Statistical analyses were performed using t-test. Significant *p*-values are (B) *p* = 0.023 and (C) *p* = 0.0087 versus asymptomatic plaques. Immunohistochemical analysis of CD68 positive macrophage cells and PGC-1α expression in human atherosclerotic plaque.Quantification of PGC-1α expression using Aperio Software Analysis System nuclear analysis algorithm.Western blot and real time PCR analysis of PGC-1α expression in symptomatic and asymptomatic plaques. Data represent the mean ± SEM of five independent experiments.

**Figure 3:**
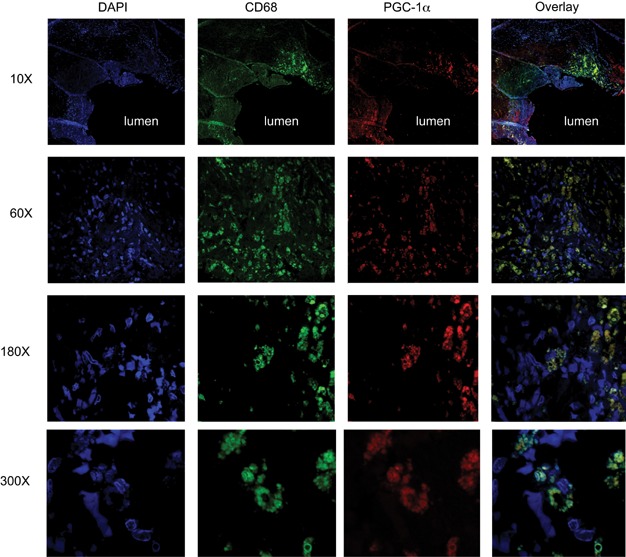
PGC-1α is expressed in macrophages in human atherosclerotic plaque Confocal microscopy at 10×, 60×, 180× and 300× shows that PGC-1α (red) is localized to CD68 macrophage positive (green) cells in human atherosclerotic plaque. Nuclei are stained with DAPI (blue). Images are representative of five independent experiments.

Atherosclerotic plaque samples were obtained from symptomatic (*n* = 5) and asymptomatic (*n* = 5) patients undergoing carotid endarterectomy. Full patient details including disease classification, lipid profile, diabetic status and medications are provided in the Online Supporting Material (Supporting Information Table S1). Immunohistochemical analysis and confocal microscopy confirmed that PGC-1α was localized to the macrophage/foam cell of human tissue (Figs 2A and 3), consistent with what was observed in the murine model. Using the ScanScope XT Digital Slides Scanner and the Aperio Software Analysis System (Nuclear Analysis Algorithm) we showed decreased PGC-1α expression in atherosclerotic plaques from symptomatic patients relative to the plaques from asymptomatic patients (Fig 2B). Furthermore, Western blotting and real time PCR analysis confirmed that PGC-1α expression is decreased in symptomatic compared with asymptomatic plaques (Fig 2C). We further scanned regions of co-localization (60×, oil) in an optimized 3D z-stack as described above (Supporting Information Movie S2). To confirm the specificity of altered PGC-1α expression in human atherosclerosis disease progression we analysed by real time PCR analysis, mRNA expression of transcription factors, known to interact with PGC-1α, in symptomatic and asymptomatic atherosclerotic plaques. Plaque RNA was standardized using total RNA content and by using 18S as a housekeeping gene to facilitate comparisons of transcripts between symptomatic and asymptomatic plaques. CT values of all genes analysed are provided in the Supporting Information (Supporting Information Table S2). PGC-1α interacts with several nuclear transcription factors, including nuclear respiratory factor (NRF)-1 and NRF-2 (Knutti & Kralli, 2001). Indeed, PGC-1α co-activation of NRF-1, promotes the expression of nuclear-encoded mitochondrial proteins (NEMP), as well as mitochondrial transcription factor A (TFAM) (Kelly & Scarpulla, 2004).

Our data shows that coincident with decreased PGC-1α expression, there was a significant decrease in expression of both *Nrf-1* and *Tfam* in symptomatic plaques compared with those obtained from asymptomatic patients. It has previously been shown that PGC-1α is a co-activator of the liver X receptor (LXR) alpha (Oberkofler et al, 2003). LXRs control the transcription of several genes involved in cellular cholesterol efflux including ABCA-1. However, in the liver LXRα down-regulates PGC-1α which is in contrast to that observed in white fat, where LXRα has no effect on expression of PGC-1α. This suggests that the effects of LXRα on PGC-1α are tissue-specific (Laffitte et al, 2003). In keeping with this, we show increased LXRα expression in plaque from symptomatic patients compared with asymptomatic patient suggesting that, similar to what was observed in the liver, LXRα and PGC-1α expression in human atherosclerotic tissue are inversely linked (Supporting Information Fig S3).

### CLA inhibits oxLDL uptake in macrophage cells

**Figure 4:**
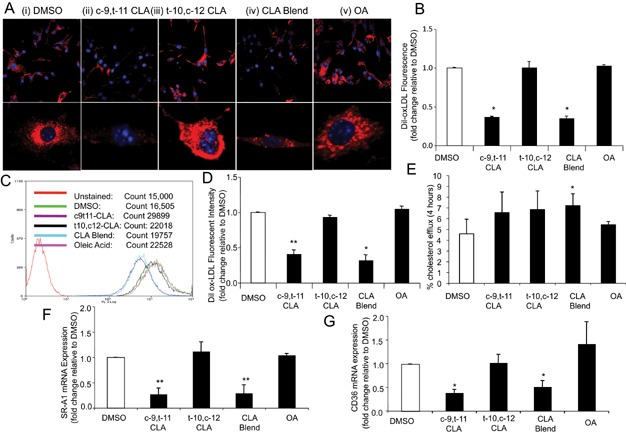
Effect of CLA on foam cell formation in RAW 264.7 macrophages RAW 264.7 macrophages were pre-treated with (i) DMSO, or 25 μM (ii) c-9,t-11-CLA (iii) t-10,c-12-CLA (iv) CLA blend (80:20 c-9,t-11:t-10,c-12-CLA) and (v) Oleic Acid (OA) for 24 h prior to ox-LDL treatment (50 μg/mL) for 4 h. Statistical analyses were performed by ANOVA. Significant *p*-values are (B) *p* = 0.027 for c9,t11CLA and *p* = 0.031 for CLA blend, (D) *p* = 0.0083 for 9,t11CLA and *p* = 0.019 for CLA blend (E) *p* = 0.049 (F) *p* = 0.0042 for c9,t11CLA and *p* = 0.0097 for CLA blend (G) *p* = 0.028 for c9,t11CLA and *p* = 0.036 for CLA blend versus DMSO control. **A.** Confocal microscopy of Dil-oxLDL (red) accumulation at 20× and 63× magnification. Nuclei are stained with DAPI (blue).**B.** Dil ox-LDL in the cells was quantified by Zeiss LSM Image Examiner.**C.** Representative flow cytometry analysis of dil ox-LDL Fluorescence signals detected at 555–600 nm.**D.** Quantification of flow cytometry analysis showing fold change in fluorescence intensity relative to DMSO.**E.** % [^3^H] cholesterol efflux of acLDL loaded RAW 264.7 macrophages towards HDL, at 4 h when compared to time zero following pre treatment with CLA isomers, CLA blend or OA.**F, G.** mRNA expression of (F) SR-A1 and (G) CD36. Data are the mean ± SEM of three independent experiments.

We next examined if CLA mediates its atheroprotective effect via altering macrophage phenotype. RAW 264.7 macrophages were pre-treated for 24 h with 25 µM of CLA isomers, CLA blend, OA or DMSO followed by 50 µg/mL Dil ox-LDL for 4 h and analysed by confocal microscopy and flow cytometry. Fluorescent intensity of Dil ox-LDL was significantly reduced in cells treated with c9,t11-CLA, CLA blend and OA relative to DMSO (Fig 4A and B). Flow cytometry confirmed that Dil ox-LDL cellular accumulation was significantly reduced in cells treated with c9,t11-CLA and CLA blend (1 vs. 0.37-fold ± 0.01, *p* = 0.0083 and 1 vs. 0.35-fold ± 0.02, *p* = 0.019, respectively) relative to DMSO (Fig 4C and D). Moreover, mRNA expression of adipophilin, a protein associated with lipid droplets in macrophage-derived foam cells (Larigauderie et al, 2004), was significantly decreased in macrophages pre-treated with c9,t11-CLA and CLA blend (Supporting Information Fig S4), consistent with the decreased lipid accumulation.

To further investigate the effect of CLA on foam cell formation, an acetylated LDL uptake assay was performed. Scintillation counts indicated that CLA isomers and CLA blend inhibit uptake of acetylated LDL compared to DMSO control (Supporting Information Table S3).

To better understand if the observed inhibition of foam cell formation is primarily due to decreased oxLDL uptake, increased cholesterol efflux or a combination of both, we examined the effect of the individual CLA isomers and CLA blend on cholesterol efflux towards HDL. Cells were left for 48 h after labelling to ensure complete uptake of 3H cholesterol. Cells were then treated in media containing 0.2% BSA for 24 h to equilibrate as previously described (Weiber et al 2009). In addition, the percentage efflux due to passive diffusion was determined and subtracted from values of that for the acceptor to definitively calculate efflux. Cholesterol efflux was calculated as the percentage of radioactivity (3H cholesterol) associated with the medium over the sum of radioactivity of both medium and lysate. Only the CLA blend showed a trend towards increased cholesterol efflux compared with DMSO (Fig 4E). This data was further supported by *in vivo* quantification of plasma cholesterol, triglycerides and lipoprotein fractions from apoE^−/−^ mice with/without supplementation. CLA supplementation had no effect on HDL, LDL, VLDL, total cholesterol or triglycerides despite inducing regression of pre-established atherosclerosis in the apoE^−/−^ model (Supporting Information Fig S5).

Together the findings suggest that inhibition of oxLDL uptake is the predominant mechanism for c9,t11-CLA and CLA blend mediated inhibition of foam cell formation. Indeed, consistent with this hypothesis, CLA significantly suppressed *Cd36* and *Sra-1* scavenger receptor expression (Fig 4F and G).

### CLA isomers induce expression of PGC-1α and downstream target genes

**Figure 5:**
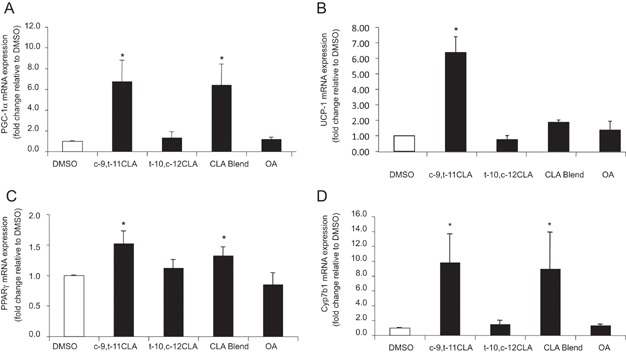
Effect of CLA on PGC-1α expression **A–D.** CLA and control lipids treatment on mRNA expression of (A) PGC-1α, (B) PPARγ1, (C) UCP-1 and (D) Cyp7b1 in oxLDL treated RAW 264.7 macrophages by real time PCR analysis. Cells were treated as described in Fig 4. Data represent the mean ± SEM of three independent experiments. Statistical analyses were performed by ANOVA. Significant *p*-values are (A) *p* = 0.028 for c9t11-CLA, *p* = 0.031 for CLA blend; (B) *p* = 0.017 for c9t11-CLA; (C) *p* = 0.043 for c9t11-CLA, *p* = 0.041 for CLA blend and (D) *p* = 0.029 for c9t11-CLA, *p* = 0.035 for CLA blend versus DMSO control.

We then investigated if CLA inhibition of foam cell formation was associated with regulation of PGC-1α expression. Initially we examined the expression of PGC-1α and its downstream target gene UCP-1 in RAW macrophages pre-treated for 24 h with 25 µM of CLA isomers, CLA blend, OA or DMSO followed by 4 h treatment with 50 µg/mL Dil ox-LDL. Real time PCR analysis was performed using 18S as housekeeping gene. CT Values for all genes are provided in Online Supporting Information (Supplementary Information Table S2). c9,t11-CLA and the CLA blend significantly increased *Pgc-1α* (1 vs. 6.78-fold ± 2.00 and 1 vs. 6.43-fold ± 1.95, *p* = 0.028 and 0.031, respectively) and induced a small but significant increase in *Pparγ* expression. In addition c9,t11-CLA increased *Ucp1* expression (1 vs. 6.38-fold ± 1.01, *p* = 0.017) whereas this was not observed with the CLA blend. Cyp7b1 is an enzyme involved in the metabolism of cholesterol to bile acid. We showed that c9,t11-CLA and CLA blend significantly increased *Cyp7b1* expression (1 vs. 8.09-fold ± 3.19, 1 vs. 7.08-fold ± 3.93, *p* = 0.029 and 0.035, respectively) suggesting that CLA may also enhance the metabolism of ox-LDL by macrophages (Fig 5). It is important to note that CLA may induce Cyp7b1 and UCP-1 through different pathways that differentiate between the two CLA isomers. Indeed, induction of UCP-1 is sensitive to only one isomer, c9,t11 CLA. The response to the blend suggests an antagonistic effect of the t10,c12 CLA isomer in the CLA blend. This is supported by a previous study, which showed isomeric differences of CLA on UCP-1. Metges et al, reported that c9,t11 CLA increased, whereas t10,c12 CLA inhibited UCP-1 mRNA expression in primary adipocytes (Metges et al, 2003).

### Overexpression of PGC-1α inhibits oxLDL uptake

**Figure 6:**
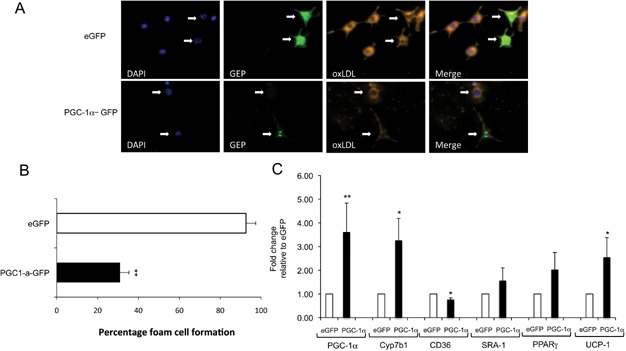
Transient transfection of PGC-1α decreases oxLDL uptake RAW 264.7 macrophages were transfected with a pEGFP-C1 vector alone (top panel) or with a pEGFP-C1 construct containing PGC-1α (lower panel) and treated with oxLDL as before. GFP positive cells are indicated by green, oxLDL is shown in yellow. Arrows indicated GFP transfected cells.Quantification of oxLDL uptake inhibition in GFP positive cells expressing PGC-1α versus control cells. Data represent the mean ± SEM of three independent experiments. Statistical analyses were performed by *t*-test, *p* = 0.0026 versus eGFP.mRNA expression of PGC-1α, Cyp7b1, CD36, SRA-1, PPARγ1 and UCP-1 in PGC-1α transfected cells compared with controls. Data represents the mean ± SEM of three independent experiments. Statistical analyses were performed by *t*-test. Significant *p*-values are as follows: *p* = 0.0082 for PGC-1α, *p* = 0.038 for Cyp7b1, *p* = 0.011 for CD36 and *p* = 0.0106 for UCP-1 versus eGFP.

In the next series of experiments, we examined whether PGC-1α directly regulates foam cell formation. RAW 264.7 macrophages were transfected with a pEGFP-C1 vector construct containing PGC-1α and treated with oxLDL as before. Confocal microscopy (Fig 6A) and quantification of foam cell formation (Fig 6B) showed that overexpression of PGC-1α resulted in a significantly lower percentage of foam cells following oxLDL treatment (30.9 ± 4.4% vs. 92.5 ± 4.8%, *p* = 0.0026) which was confirmed by timelapse imaging (Supporting Information Video S3). Furthermore, transfection of GFP-PGC-1α significantly increased *Pgc-1α*, *Cyp7b* and *Ucp1* expression and induced a significant decrease in *Cd36* expression compared to empty vectors. There was however, no change in *Sra*-1 expression. Similar to what was observed in CLA treated macrophages, there was a modest increase in PPARγ expression (Fig 6C). Although inhibition of foam cell formation is clearly visualized in PGC-1α over expressing cells, it is important to note that the effects of PGC-1α over expression on gene regulation may be confounded as a result of low transfection efficiency. CT values of all genes analysed are provided in Online Supplementary Information (Supplementary Information Table S2).

PGC-1α is involved in modulation of radical oxygen species (ROS) formation. It has previously been shown that overexpression of PGC-1α decreases ROS in human smooth muscle and endothelial cells (Kim et al, 2007). To investigate this we measured intracellular ROS generation in RAW macrophages transfected with 0.25 and 0.5 µg PGC-1α-GFP or with control eGFP empty vector and treated with oxLDL as previously described (Mohanty et al, 1997). Overexpression of PGC-1α has no effect on ROS in RAW macrophage cells when compared with empty vector (Supporting Information Fig S6) suggesting that modulation of ROS is not the mechanism through which PGC-1α limits foam cell formation. Indeed this further supports our hypothesis that inhibition of uptake of oxLDL is the primary mechanism through which PGC-1α inhibits foam cell formation.

### oxLDL uptake is increased in BMDMs from PGC-1α^−/−^ animals

**Figure 7:**
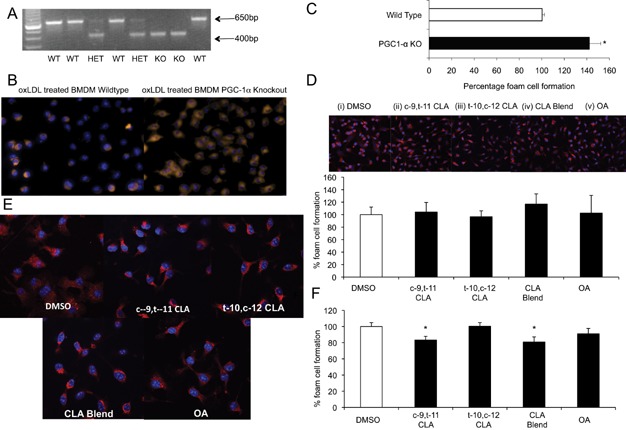
PGC-1α gene deletion increases foam cell formation in bone marrow derived macrophages **A.** PCR products of genomic DNA from PGC-1α^−/−^ (KO, 650-bp), PGC-1α^+/+^ (WT, 400-bp) and PGC-1α^+/−^ animals.**B, C.** (B) Fluorescence microscopy with oxLDL in yellow and DAPI stained nuclei in blue and (C) Quantification of foam cell formation in BMDMs from WT and KO mice treated with 50 μg oxLDL. Data represents the mean ± SEM of three independent experiments. Statistical analyses were performed by *t*-test, *p* = 0.032 versus wildtype.**D, E.** (D) Confocal microscopy at 20× and quantification of foam cell formation in BMDMs from PGC-1α^−/−^ mice pre-treated with 25 μM CLA isomers, CLA blend and OA followed by oxLDL (shown in red) and (E) Confocal microscopy at 63× of foam cell formation in BMDMs from PGC-1α^−/−^ mice pre-treated with CLA isomers and controls. Nuclei are stained with DAPI (blue).**F.** Quantification of foam cell formation in BMDMs from PGC-1α^+/+^ mice pre-treated with 25 μM CLA isomers, CLA blend and OA followed by oxLDL. Data represents the mean ± SEM of three independent experiments. Statistical analyses were performed by *t*-test, *p* = 0.039 for c9t11CLA and *p* = 0.041 for CLA blend versus DMSO.

In further experiments, we examined the effect of PGC-1α in foam cell formation by exploring the impact of gene deletion on oxLDL uptake in BMDMs from WT and PGC-1α^−/−^ mice (Fig 7A). BMDM deletion of PGC-1α from KO mice was confirmed by Western blotting (Supporting Information Fig S7). BMDMs were treated for 4 h with 50 μM oxLDL and analysed by fluorescence microscopy. In the absence of PGC-1α, there was a marked increase in oxLDL accumulation, this was confirmed by quantitative analysis, which showed significantly greater foam cell formation in BMDMs from PGC-1α^−/−^ mice compared with those from WT mice (142.0 ± 10.3% vs. 100 ± 2.31%, *p* = 0.032) (Fig 7B and C).

To investigate if the induction of PGC-1α is required for the protective effects of CLA on cholesterol uptake and foam cell formation, BMDMs from PGC-1α^−/−^ mice were treated with DMSO, OA, CLA isomers or CLA blend for 24 h prior to treatment with oxLDL. Using confocal microscopy we showed that in the absence of macrophage PGC-1α, CLA fails to inhibit foam cell formation (Fig 7D and E). This is in contrast to the effect of CLA isomers on foam cell formation in BMDMs from wildtype animals where as expected both c9t11 CLA and CLA blend inhibited foam cell formation (Fig 7F). These data confirm a role for PGC-1α in the inhibition of foam cell formation by CLA.

### Macrophage deletion of PGC-1α^−/−^ increases atherosclerotic lesion formation *in vivo*

In a murine model of atherosclerosis developed in LDLR^−/−^ mice fed a high cholesterol diet for 4 months, peritoneal macrophages display features of foam cells (Supporting Information Fig S8A). In these cells, we showed decreased expression of PGC-1α protein and mRNA expression (Supporting Information Fig S8B and C). Further analysis of aortas from normal and hypercholesterolemic LDLR^−/−^ mice showed that PGC-1α mRNA expression is also decreased in aortas that contain extensive atherosclerotic lesions (Supporting Information Fig S8D). To examine if PGC-1α is a causal gene in atherosclerosis we initially examined the effect of a high cholesterol supplementation on serum cholesterol and triglycerides and on lesion formation in PGC-1^−/−^ mice. PGC-1^+/+^ (*n* = 10) and PGC-1^−/−^ (*n* = 10) were administered a 1% cholesterol chow for 16 (*n* = 5 in each group) or 20 weeks (*n* = 5) in each group. Following 16 or 20 weeks on a 1% cholesterol diet there was no difference in serum cholesterol or triglycerides between PGC-1α^+/+^ and PGC-1α^−/−^ mice (Supporting Information Fig S9A). At the time of sacrifice the aorta, including the root, arch and thoracic lumbar regions, were carefully dissected from all mice. *En face* analysis of the aortic arch showed that global deletion of PGC-1α^−/−^ had no effect on atherosclerosis development (Supporting Information Fig S9B). Indeed there was no evidence of atherosclerotic plaque formation or lipid deposition in either the aortic arch or aortic root regardless of the genotype or duration of high cholesterol supplementation.

**Figure 8:**
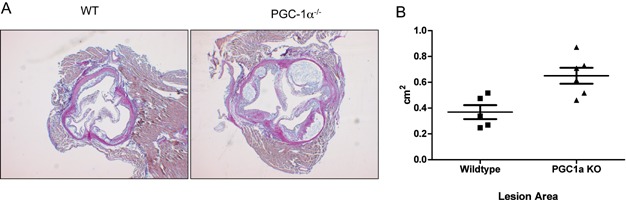
Macrophage PGC-1α deficiency increases atherosclerotic lesion formation in the LDLR^−/−^ mouse **A, B.** (A) Representative images of atherosclerotic lesion formation and (B) Quantification of atherosclerosis lesion formation in LDLR^−/−^ mice transplanted with Wildtype (WT) and PGC-1α^−/−^ (KO) bone marrow cells administered a high cholesterol diet for 4 months. PGC-1α^−/−^ transplanted mice show a significant increase in the size of atherosclerotic lesions. Data represent the mean ± SEM of five independent experiments. Statistical analyses were performed by Mann-Whitney test, *p* = 0.0177.

This was not surprising in the context of a previous study by Stein et al, which investigated the effect of total PGC-1α deficiency in an atherosclerotic mouse model (Stein et al, 2010). That study showed that there was no increase in atherosclerotic lesion formation or plaque burden in apoE^−/−^/PGC-1α^−/−^ mice compared with their apoE^−/−^/PGC-1α^+/+^ littermates. However, there were more CD68-positive macrophages and ICAM-1-expressing cells in plaques from apoE^−/−^/PGC-1α^−/−^ mice compared to those from littermate controls, implicating a potential role of macrophage PGC-1α in atherosclerosis *in vivo* which is in keeping with the data presented above. Therefore, we hypothesized that macrophage PGC-1α may play a role in the pathogenesis of the disease. Thus, to definitively identify a role for PGC-1α in atheroprotection we performed bone marrow reconstitution experiments. Irradiated LDLR^−/−^ mice were reconstituted with PGC1α^−/−^ or WT bone marrow cells. After 4 months on a high cholesterol chow, the extent of atherosclerosis was quantified. PGC1α^−/−^ transplanted mice show a significant increase in the size of atherosclerotic lesions (Fig 8). These novel data show that macrophage PGC-1α plays an important role in atheroprotection.

## DISCUSSION

A critical step in the development of atherosclerosis is the accumulation of cholesterol in macrophages, which leads to foam cell formation. Cellular cholesterol content in macrophages is determined by uptake (mediated by scavenger receptors) and efflux of cholesterol (mediated by cholesterol acceptors) (de Villiers & Smart, 1999), an imbalance of which results in the formation of foam cells, which in turn promote lipid deposition and lesion growth.

CLA both inhibits progression (Lee et al, 1994) and induces regression (Toomey et al, 2006) of atherosclerosis in animal models. Here we provide evidence that the c9,t11-CLA isomer and the atheroprotective CLA blend alter the phenotype of the macrophage cell by inhibiting oxLDL uptake and subsequent foam cell formation, which confirm previous findings showing that CLA treatment of RAW 264.7 cells regulates foam cell formation (Ringseis et al, 2008). Interestingly, t10,c12-CLA had no effect on foam cell formation, consistent with the divergent effects of the two CLA isomers in atherosclerosis (Arbonés-Mainar et al, 2006). To determine if CLA inhibited oxLDL uptake, increased cholesterol efflux or a combination of both, we examined cholesterol efflux in acLDL loaded macrophage cells. Only the CLA blend altered cholesterol efflux towards HDL suggesting that inhibition of cholesterol uptake is the primary mechanism of action of CLA isomers in inhibiting foam cell formation. However, the increased efflux is consistent with the increased expression of ABCA1 in murine aorta during CLA-induced regression and thus it is feasible to suggest that this may be also related to the increase in Cyp7b1 as a mechanism for hydroxylated sterol removal. SR-A1 and CD36 have been identified as the two major receptors responsible for lipoprotein uptake into macrophages and mice lacking either receptor show a reduction in atherosclerotic lesions (Suzuki et al, 1997; Febbraio et al, 2000). We show reduced expression of the scavenger receptors, confirming a role for CLA in inhibition of oxLDL uptake.

One possible mechanism for the effects of CLA is activation of PPARγ (Ringseis et al, 2006). In the PPARγ knockout model, there is a reduction in oxLDL accumulation in macrophages but no difference in uptake of oxLDL (Babaev et al, 2005). In parallel PPARγ agonists do not change SRA-1 expression but upregulate the expression of CD36 (Tontonoz et al, 1998). In contrast, we found that CLA inhibits both CD36 and SRA-1 suggesting that PPARγ activation does not fully explain the effects of CLA. Indeed, we have previously shown that CLA inhibits monocyte migration towards platelet releasate through a PPARγ- independent mechanism (McClelland et al, 2010).

Using a transcriptomic approach we looked for additional related or distinct genes/pathways regulated by CLA that may yield further information as to how CLA modulates atherosclerosis and/or macrophage function. In searching for potential targets we identified the nuclear receptor co-activator PGC-1α, as a nexus gene in a network regulated by CLA. Interestingly, CLA regulation of PGC-1α has previously been reported in chronic inflammation. Bassaganya-Riera et al showed that CLA ameoliorated colitis in an experimental model. Interestingly, CLA induced both PPARγ and PPARδ expression and transcriptionally regulated gene clusters involved in lipid metabolism including UCP1, PGC-1α and CD36 which is in keeping with our findings (Bassaganya-Riera et al, 2004). Indeed, several activities of PGC-1α suggest it could play a role in atherosclerosis (Oberkofler et al, 2003; Qu et al, 2009; Xu et al, 2010; Zhang et al, 2007).

To validate the CLA regulation of PGC-1α *in vivo* we investigated the expression of PGC-1α in the CLA-induced regression model of atherosclerosis (Toomey et al, 2006). There was increased PGC-1α mRNA and protein expression which was localized to the macrophage cell in the aorta of apoE^−/−^ mice supplemented with CLA coincident with a significant increase in expression of the PGC-1 α target gene, UCP-1. Interestingly, there was also decreased macrophage PGC-1α expression in plaques from symptomatic compared to asymptomatic patients, consistent with a role for PGC-1α in regulating the severity of disease. This is the first report that has identified dysregulation of PGC-1α in macrophage/foam cell in murine and human atherosclerosis.

We investigated the expression of PGC-1α in CLA mediated inhibition of foam cell formation. It is important to note that to date there has been no direct evidence linking PGC-1α with foam cell formation, although recently Stein et al reported increased accumulation of CD68 positive macrophage cells and ICAM-1 expression in atherosclerotic plaques from apoE^−/−^/PGC-1α^−/−^ DKO mice when compared with apoE^−/−^ mice (Stein et al, 2010). The data presented here show that c9-t11 CLA and CLA blend, which inhibit foam cell formation, also induce the expression of PGC-1α in oxLDL loaded macrophages.

To further address a role of PGC-1α in atheroprotection we investigated oxLDL accumulation in macrophage cells transfected with PGC-1α. Although not all cells expressed the GFP plasmid, those that did maintained the macrophage phenotype and did not accumulate oxLDL. Furthermore, similar to what was observed in RAW macrophages treated with CLA, over expression of PGC-1α significantly increased the expression of Cyp7b1 and UCP-1 and induced a small but significant decrease in *Cd36* expression although this was not of the same magnitude as that observed with CLA treatment likely due to the low transfection efficiency of the cells. We supported these data by examining the effect of PGC-1α deletion on oxLDL uptake in primary bone marrow derived macrophages from PGC-1α^−/−^ animals. Our data shows that PGC-1α gene deletion increases foam cell formation. Importantly, treatment of BMDMs from PGC-1α KO animals treated with CLA isomers or CLA blend had no effect on foam cell formation. In the LDLR^−/−^ model of atherosclerosis peritoneal macrophages, which display characteristics of foam cells, have decreased PGC-1α expression. Importantly, using bone marrow transplantation studies we show that macrophage deletion of PGC-1α accelerated atherosclerosis and increased lesion size in the LDLR^−/−^ mouse. A limitation of our study is that the evidence for the role of macrophage PGC-1α in atherosclerosis is based primarily on bone marrow transfer studies. Therefore, additional studies for verification are required in a macrophage cell specific knockout to fully define the role of macrophage PGC-1α in this context. Nonetheless, these data for the first time describe a role for PGC-1α in macrophage function and regulation of foam cell formation and moreover, suggest that there is an endogenous pathway involving PGC-1α that can be activated, for example by CLA, to limit, or induce regression of, atherosclerosis.

PGC-1α expression and/or CLA may invoke additional anti-atherogenic pathways such as the metabolism of cholesterol to bile acids.

In both CLA treated macrophages and in murine aorta in the CLA-induced regression model, we have shown an increase in Cyp7b1 expression, which is located in extrahepatic tissue (Chiang, 2002) where it inactivates oxysterols, products generated during the breakdown of oxLDL (Setchell et al, 1998). Enzymes involved in cholesterol to bile acid synthesis, specifically Cyp27, have been implicated to play a role in atherosclerosis (Björkhem et al, 1994) and thus it is feasible to suggest that increased Cyp7b1 expression by CLA, increases the removal of cholesterol metabolites from foam cells resulting in a less lipid laden foam cell phenotype. In our transcriptomic analysis we identified the nuclear receptor RORα on the PGC1α network and confirmed its regulation in CLA-induced regression *in vivo*. It is likely that CLA mediated regulation of both PGC-1α and RORα may regulate Cyb7b1 since it has previously been shown that in RORα^−/−^ mice, which develop severe atherosclerosis, there is suppression of Cyp7b1 expression suggesting RORα may be a positive regulator of Cyp7b1 (Wada et al, 2008).

Several studies provide clues to the mechanism of PGC-1α regulation by CLA. Activation of PPARγ in adipocyte cells induces PGC-1α expression due to a PPRE at – 2043–2055 in the distal promoter sequence of mouse PGC-1α (Hondares et al, 2006) and here we show increased PPARγ expression in CLA treated macrophages. An alternative pathway is through activation of AMPK. Treatment of primary myotubes with the AMPK agonist AICAR, results in direct phosphorylation of the PGC-1α protein at threonine 177 and serine 538 (Jäger et al, 2007). We have recently shown an increase in phosphorylated/activated AMPK in aortic samples from CLA fed mice compared to cholesterol fed mice (Supporting Information Fig S10), suggesting that activation of AMPK may underlie the CLA regulation of PGC-1α.

Our work with CLA is designed to identify pathways that may prevent the progression and/or induce the regression of atherosclerosis. In this study we demonstrate that expression of PGC-1α is increased during CLA-induced regression of atherosclerosis and that CLA inhibition of foam cell formation is linked to induction of PGC-1α and that the gene alone could inhibit foam cell formation. Furthermore, we show that macrophage specific deletion of PGC-1α increases atherosclerosis *in vivo*. Importantly, we showed that PGC1α is expressed in the plaques of patients, raising the possibility that this is a regulatory pathway in human atherosclerosis.

## MATERIALS AND METHODS

### Animals

All animal experiments were conducted in conformity with International laws and policies. A license and permission for the study were obtained from the Department of Health.

An 80:20 isomeric blend of the c9,t11:t10,c12-CLA isomers, was incorporated into 1% cholesterol chow (Special Dietary Services, Essex, UK). ApoE^−/−^ mice (*n* = 40) received a 1% cholesterol chow for 8 weeks. After 8 weeks, the animals were either continued on the 1% cholesterol diet for a further 4 weeks (*n* = 20) or continued on a 1% cholesterol diet supplemented with 1% CLA blend for 4 weeks (*n* = 20). PGC-1α^−/−^ and PGC-1α^+/+^ mice received a 1% cholesterol chow for 16 (*n* = 5 per group) or 20 (*n* = 5 per group) weeks. Affymetrix gene arrays (Mouse 430 2.0) (*n* = 4 per group) were performed on RNA isolated from aortic tissue. Confocal microscopy was performed on 5 µm serial paraffin-embedded aortic sections using the Vectastain Universal Kit (Vector Laboratories, UK). Sections were incubated with PGC1-α (Alexa Fluor 568 goat anti-rabbit) and F4/80 (Alex Fluor 647 rat-anti-mouse Invitrogen, USA) at a 1:200 dilution. Sections were counterstained with 4,6-diamidino-2-phenylindole dihydrochloride (DAPI) (10 µg/mL) to identify nuclei and slides were analysed with Olympus FV1000 confocal microscope. Immunohistochemistry, Western blotting and real time PCR analysis of aorta was performed using previously described methods (Toomey et al, 2006) and as described in the online Supporting Information. Total plasma cholesterol and triglycerides were measured using Cholesterol CHOD-PAP and Triglycerides GPO-PAP kits respectively (Roche, Mannheim, Germany) and as described in detail in the online Supplementary Information.

**The paper explained**

PROBLEM:

Atherosclerosis, the primary cause of heart disease and stroke is a complex progressive disease. In addition to the role of lipids, atherosclerosis has many of the features of an inflammatory disease including infiltration of inflammatory cells such as monocytes and macrophages. Despite the information on the pathways and mechanisms involved in the development of atherosclerosis, there have been no defined pathway(s) which would explain how atherosclerosis could be reversed. The implications of this are important since most patients present with pre-established lesions and the therapeutic goal would be to reverse the lesion. We have previously shown that dietary administration of conjugated linoleic acid (CLA) induces regression of pre-established atherosclerosis in the apoE^−/−^ mouse model. Understanding the mechanisms involved through which CLA mediates regression may help identify pathways that limit or reverse human atherosclerosis.

RESULTS:

In a transcriptomic screen of genes regulated during CLA-mediated regression of atherosclerosis, we identified several gene clusters, one of which contained the gene PGC-1α at its hub. Given the role of PGC-1α in regulating genes involved in lipid metabolism and its emerging role in vascular cell function we pursued PGC-1α as a potential target of CLA induced atherosclerosis regression. The data presented show that expression of PGC-1α is increased during CLA-induced regression of murine atherosclerosis in vivo. Expression of PGC-1α was localized to macrophage/foam cells in murine and human atherosclerosis. Interestingly, macrophage expression of PGC-1α was inversely linked with disease progression in patients with the disease. The functional significance of macrophage PGC-1α was also addressed. Deletion of PGC-1α in bone marrow derived macrophages promoted foam cell formation, whilst over expression of the gene inhibited foam cell formation. Significantly, macrophage specific deletion of PGC-1α accelerated atherosclerotic lesion formation *in vivo*.

IMPACT:

Our work with CLA is designed to identify pathways that may prevent the progression and/or induce the regression of atherosclerosis. In this study we demonstrate that CLA inhibition of foam cell formation is linked to induction of PGC-1α and that the gene alone could inhibit foam cell formation. Furthermore, we show that macrophage specific deletion of PGC-1α increases atherosclerosis *in vivo*. Importantly, we showed that PGC1α is expressed in the plaques of patients, raising the possibility that this is a regulatory pathway in human atherosclerosis.

### Immunhistochemical analysis of human atherosclerotic plaque

The study was approved by the Ethics Committee of St. Vincent's University Hospital, Dublin Ireland. All patients gave written informed consent. Ten patients (mean age 67 ± 13 years, 8 males and 2 females) with clinical and angiographic evidence of atherosclerosis were studied. All patients were undergoing carotid endarterectomy. Symptomatic plaques [defined as patients who had had a first-ever Transient Ischemic Attack (TIA) or non-disabling ischemic stroke from atherosclerotic stenosis and without any known history or diagnosis of CVD]; and asymptomatic plaques (defined as those from patients without any previous ischemic events or known CVD) were obtained. All specimens were immunostained using a polyclonal Rabbit Anti-Human-PGC-1α (Calbiochem) and a Monoclonal Mouse Anti-Human-CD-68 (Dako), respectively. After acquisition of Digital Scanned Slides with the ScanScope XT Scanner (Aperio). The Aperio Software Analysis System *Nuclear Analysis* algorithm was applied to quantify the level of PGC-1α expression per cell and then the total of PGC-1α positive cells per tissue section. Prior to this evaluation, a calibration of the immunostaining for DAB and Haematoxylin staining was required and performed using *Colour Deconvolution* algorithm, which provided a threshold level of PGC-1α expression. Nuclear analysis was then carried out on stained sections to establish the target protein expression firstly, as ‘expression per cell’, and then as ‘total per tissue section’. This algorithm evaluated the intensity of DAB staining (protein staining) in each haematoxylin (nuclei) stained cell and specifically and unequivocally assigned a mark-up image composed of different colours, corresponding with different levels of protein expression, which was counted and quantified. The algorithm used is outlined in the Supporting Information (Supporting Information Fig S11).

Colocalization of PGC-1α to macrophage cell was performed by confocal microscopy as described above, using an Alexa Fluor 647 goat anti-rabbit IgG (H + L) (Invitrogen), for PGC-1α, and an Alexa Fluor 594 goat anti-mouse IgG F(ab′)_2_ fragment (Invitrogen), for CD-68.

### Foam cell formation

RAW 264.7 macrophages were treated for 24 h with 25 µM c9,t11-CLA, t10,c12-CLA, CLA blend (80:20 c-9t,11-CLA: and t10,c12-CLA), OA or DMSO. Cells were exposed to 50 µg/mL of either non-labelled ox-LDL, or Dil labelled RP-177 (Dil ox-LDL) for 4 h. RAW 264.7 were transfected with 0.5 μg of a pEGFP-C1 vector construct containing PGC-1α (Addgene) using Fugene HD transfection reagent (Invitrogen), for 24 h. Cells were treated with 50 μg/mL Dil oxLDL for 4 h and visualized by confocal microscopy and flow cytometry (Coulter EPICS XL-MCL). Details of the RNA isolation, primer sequences and flow cytometry methods are described in the online Supporting Information.

### Cholesterol uptake and efflux assays

Cholesterol uptake assays were performed as previously described (Chinetti et al, 2001). Details are provided in the online Supporting Information. To investigate cholesterol efflux, Raw 267.4 Macrophages were loaded with AcLDL prior to addition of extracellular cholesterol acceptors as described previously (Weibel et al, 2009). Briefly, after [^3^H] cholesterol labelling the cells, media containing HDL (20 µg/mL) or media alone was added for up to 4 h, filtered and counted by liquid scintillation.

### Bone marrow transplantation

Irradiated LDLR^−/−^ mice were reconstituted with PGC1α^−/−^ or WT macrophages. Mice were then placed on a high fat (HF) diet (0.06% added cholesterol and 21% milk fat) for 4 months. Atherosclerosis was quantified by computer-assisted image analysis in cross-sections through the aortic origin, as previously described (Li et al, 2000).

### Data analysis

Microarray analysis was performed with replicates using linear modelling with empirical Bayes framework to determine differential expression. This generated moderated t-statistics and we used *p* < 0.01 after correction for multiple hypothesis testing using the Benjamini & Hochberg method. A 1.7-fold cut-off was applied to detect significant differences in expression between the two study groups. To elucidate the important molecular and cellular networks represented by genes using the 1.7-fold cut-off, we applied unbiased pathway analysis using IPA.

### Statistical analysis

Results are expressed as mean ± SEM. Experimental points were performed in triplicate with a minimum of three independent experiments. Statistical comparisons between DMSO versus treated groups were made by Student's unpaired *t*-test or ANOVA. A value of **p ≤ 0.01 and *p ≤ 0.05 were considered significant.

## For more information

International Atherosclerosis Society:

http://www.athero.org/

American Heart Association Council on Atherothrosclerosis Thrombosis and Vascular Biology:

http://my.americanheart.org/professional/Councils/ATVB/Council-on-Arteriosclerosis-Thrombosis-and-Vascular-Biology_UCM_322848_SubHomePage.jsp

Belton Laboratory at University College Dublin

http://www.ucd.ie/research/people/biomolecularbiomedscience/drorinabelton/

## Supplementary material

As a service to our authors and readers, this journal provides supporting information supplied by the authors. Such materials are peer reviewed and may be re-organized for online delivery, but are not copy-edited or typeset. Technical support issues arising from supporting information (other than missing files) should be addressed to the authors.
