# Supplementary material for: Macrophage PPAR gamma Co-activator-1 alpha participates in repressing foam cell formation and atherosclerosis in response to conjugated linoleic acid
Source: EMBO Mol Med. 2013 Aug 21;5(9):1443–57. doi: 10.1002/emmm.201302587 (PMC3799497; doi:10.1002/emmm.201302587)
Supplement: Supplementary file 1 [file emmm0005-1443-SD1.pdf]

EMBO Molecular Medicine

Macrophage PPAR gamma Co-activator-1 alpha participates in repressing foam cell formation and atherosclerosis in response to conjugated linoleic acid.

Cathal McCarthy, Nora T. Lieggi, Denis Barry, Declan Mooney, Monica de Gaetano, William G. James, Sarah McClelland, Mary C. Barry, Laure Escoubet-Lozach, Andrew C. Li, Christopher K. Glass, Desmond J. Fitzgerald and Orina Belton

Corresponding author: Orina Belton, University College Dublin

Review timeline:

Submission date:	01 February 2013
Editorial Decision:	04 March 2013
Revision received:	05 May 2013
Editorial Decision:	17 June 2013
Revision received:	27 July 2013
Accepted:	02 July 2013

Transaction Report:

(Note: With the exception of the correction of typographical or spelling errors that could be a source of ambiguity, letters and reports are not edited. The original formatting of letters and referee reports may not be reflected in this compilation.)

Editor: Roberto Buccione

1st Editorial Decision 04 March 2013

Thank you for the submission of your manuscript to EMBO Molecular Medicine. We have now received reports from the three Reviewers whom we asked to evaluate your manuscript.

You will see that while the Reviewers are generally supportive, two Reviewers raise critical points that question the impact and conclusiveness of the results, thus preventing us from considering publication at this time.

Reviewer 1 is concerned that essential experiments are missing in the manuscript. Specifically s/he would like to see the effects of conjugated linoleic acid (CLA) on high cholesterol fed (or alternatively ApoE-/- mice-crossed) PGC-1a-/- mice compared to controls. Reviewer 1 lists a number of other important issues, all of which require your action.

Reviewer 2 notes that a key experiment addressing the effect of CLA on foam cell formation or progression of atherosclerosis in PGC-1a -/- mice is missing and must be performed. Reviewer 2 also raises other important issues, which require your action.

Reviewer 3 is more supportive and mentions a number of items that need to be addressed.

While publication of the paper cannot be considered at this stage, we would be prepared to consider a suitably revised submission, with the understanding that the Reviewers' concerns must be fully addressed with additional experimental data where appropriate and that acceptance of the manuscript will entail a second round of review.

Please note that it is EMBO Molecular Medicine policy to allow a single round of revision only and that, therefore, acceptance or rejection of the manuscript will depend on the completeness of your responses included in the next, final version of the manuscript.

As you know, EMBO Molecular Medicine has a "scooping protection" policy, whereby similar findings that are published by others during review or revision are not a criterion for rejection. However, I do ask you to get in touch with us after three months if you have not completed your revision, to update us on the status. Please also contact us as soon as possible if similar work is published elsewhere.

I look forward to seeing a revised form of your manuscript as soon as possible.

***** Reviewer's comments *****

Referee #1 (Comments on Novelty/Model System):

I have struggled a bit when reviewing this paper. In general, the quality of the data aquisition and presentation including additional controls and statistics, which appears to be amenable to improvement. However, the authors should in addition perform some essential experiments in PGC-1a-deficient mice by cholesterol feeding or crossing them into an Apoe-/- background.
Should these experiments be provided with adequate results, the paper may become acceptable for publication.

Referee #1 (Remarks):

Belton et al.: Macrophage PPAR gamma Co-activator-1 alpha participates in repressing foam cell formation and atherosclerosis in response to conjugated linoleic acid.

The authors intended to further clarify the mechanism of CLA-induced AS regression in cholesterol-fed Apoe-/- mice. Their lab previously demonstrated reduced macrophage infiltration in lesions and reduced adhesion molecule, MMP-9 and pro-inflammatory cytokine expression by CLAs, while Ringseis et al. reported reduced lipid accumulation and enhanced efflux.

By differential expression screening in aortic extracts from cholesterol- and +/- CLA-fed Apoe-/- mice, PGC-1a emerged as the hub of a candidate gene cluster. Subsequent experiments aimed at pinpointing the site of PGC-1a involvement into lesion macrophages and to relate it to human plaque samples.

It seems that the authors have somewhat made a second step before the first one: The PGC-1a-deficient mice are available and (on standard chow) atherosclerosis is obviously not a prominent feature of their phenotype. So why did they not first study cholesterol feeding in PGC-1a-/- mice or cross them with Apoe-/- mice (see Stein et al. 2010) before the bone marrow transplantation experiments? In these mice and their PG-1a +/+ controls the effect of CLAs and its mediation by PGC1a could have been tested directly in vivo.

Specific points:

p.5: Cyp7A1 initiates the classical (not the alternative) bile acid pathway.

p.7, design: The 1% CLA supplement was only contrasted to ´no supplement´. An additional control e.g. by an OA or LA supplement might have been of interest, although both fatty acids are not inert. LA shares the same caloric load, the same degree of unsaturation (even the same n-6, 12c-configuration with the t10,c12-CLA isomer) and covers direct PPARgamma effects.

Please mention how the aortic RNA extracts used for differential expression analysis were standardized (by wet weight / total RNA content / a house-keeping gene / internal standard etc.)?

p. 8, top: How many of the n=10 endartierectomy patients were symptomatic or asymptomatic. Their potentially confounding medication (statins, fibrates, ASA, NSAIDs, antidiabetics, antioxidants, smoking habits) and lipid profiles must be given.

p.8, bottom: Please mention, how the RNA extraction from plaques and the PCR was standardized to allow quantitative comparisons of asymptomatic vs. symptomatic plaques. Among the PGC-1a interacting transcription factors LXR is missing, which would be of special interest in the setting of regression of AS.

p.9, bottom: The efflux (Fig. 3E) assay should be described in more detail as the various fatty acids influenced lipid uptake (Fig. 3, A-D): How was equal 3H-cholesterol labelling of cells assured? How was efflux espressed (% secreted of accumulated intracellular label / % of total 3H-cholesterol added for labelling)? As HDL and apo A1 were used as acceptors please show both data. The interpretation of absence or presence of significance in efflux data should be reconsidered (n=3, large SEMs, multiple t-testing ?).

p.10 and Fig. 4: Why were RAW cells first oxLDL-loaded before exposure to CLAs? OxLDL loading provides a complex mixture of bioactive lipids and will per se stimulate PPARgamma and LXR, so effects of CLAs on PPARg and LXR might be obscured.

In addition to expression relative to DMSO control, which was set to 1 for each transcript, the relative expression to a common house keeping gene should be given to provide an estimate of the absolute expression levels (e.g. Cyp7b1 should be rather low in macrophages).

Fig. 4B: UCP-1 was stimulated by c9,711-CLA but not by the blend containing 90 c9,t11-CLA (in contrast to PGC-1a and Cyp7b1). This should be discussed.

p.11 bottom and Fig. 5A: Only two weakly PGC-1a-GFP expressing cells are shown in the small insert. Please provide an overview (like in Fig. 3 A) showing more PGC-1a-GFP+ and possibly simultaneously GFP-negative cells (like in the vector controls) to allow comparison of ox-LDL-accumulation in these cells.

p. 11 and Fig. 5C: As PGC-1a also activates LXR (Oberkofler et al.) and LXR induces ABCA1, their mRNA levels should also be analysed in PGC-1a over-expressing cells. Discuss the dramatic reduction of foam cells in the light of the minor effect on CD36 and the increase in SRA-1.
A measure of the absolute abundance of the various transcripts should be given.

p. 11/12 and Fig. 6D: The % foam cells from wt mice somewhat arbitrarily set to 100%, resulting in ´140 %´(?) in PGC1-a-KO? Rather use the same quantification as in PG1a-OE (Fig. 5B).
Fig. 6E: The technical quality of the micrographs is insufficient to allow comparison the effects.

p.12 bottom:
Why were CLA supplements not tested in BM-reconstituted LDLR-/- mice?

Discussion:
p. 14, top: ...CLA inhibits both CD36 and SRA-1... but PCG-1a-GFP transfection did not (Fig. 5 C). This should be discussed.

p.16: Björkem et al. established Cyp27, not Cyp71b, as a major player in macrophage cholesterol handling.

p. 21: Statistics: t-test is not adequate for data shown in Fig.3 A to G and Fig. 4A-D.

All the information of the two-column panels of Fig. 1B-C (with control set to 1) can be presented on much less space.

All n = x, now mentioned in methods p.18 ff., should better be given in legends to figures.

Referee #2 (Remarks):

Summary: Paper follows up on previous observations that conjugated linoleic acid (CLA) regresses atherosclerotic plaque in cholesterol-fed mice. Transcriptional analysis of atherosclerotic plaque from CLA-fed mice implicates PGC1alpha as a candidate mediating the effect of CLA on regression. Human atherosclerotic plaque was shown to have increased expression of PGC1alpha. Studies in mice showed that overexpression of PGC1alpha was associated with reduced foam cell formation in an in vitro assay while knockout was associated with an increase. Macrophage specific deletion of PGC1a in an athero-susceptible mouse model was associated with increased aortic plaque area. The authors conclude that PGC1alpha plays a role in atheroprotection.

Comments:

1) The manuscript identifies PGC1alpha as a potential driver of the anti-atherosclerotic effect of CLA. While evidence is presented that demonstrates that lack of PGC1alpha influences foam cell formation and atherosclerosis a key experiment is missing showing that CLA has no effect on foam cell formation or progression/regression of atherosclerosis in PGC1alpha knockout mice. Ideally this would be done by measuring atherosclerosis development in Ldlr knockout mice (or apoE knockout) with bone marrow transplant with cells from Pgc1alpha knockout mice with and without 1% CLA diet. If the hypothesis is correct there should be no effect of CLA on atherosclerosis in these mice.

2) The uptake of oxidized LDL in BMDMs (figure 6E) appears to be much less that what was seen using RAW cells (figure 2A). Are there any differences in oxidized LDL uptake in BMDMs from wild type of apoE knockout mice incubated with and without CLA? This should be tested.

3) The data in supplemental figure 3A showing PGC1alpha expression in human plaque is useful in demonstrating reduced PGC1alpha expression in plaque versus relative disease-free tissue. However, the comparison showing expression of PGC1alpha in plaque from symptomatic versus asymptomatic patients is dependent on the assumption that the plaque burden in symptomatic patients is greater than that in asymptomatic patients which may or may not be the case. Please delete figure 2C and replace with supplemental figure 3A.

4)Figure 3E. The cholesterol efflux assay was done using HDL and apoA-I as acceptors. Which is being shown in this figure? Efflux to both HDL and apoA-I should be reported. There appears to be increased efflux for cells treated with c-9,t-11 CLA and t-10,c-12. The lack of significance of seems to be due more to experimental variability than anything else. Increased efflux would be consistent with the increase in Abca1 shown in figure 1B and may be related to the increase in Cyp7b1 since this would be a mechanism for hydroxylated sterol removal. Please comment in the Discussion on the possibility that increased efflux contributes the reduced foam cell formation seen in response to CLA.

5) Page 9. Cholesterol uptake assay is actually an acetylated LDL uptake assay using a tritiated cholesterol label. Please change to cholesterol uptake assay to acetylated LDL uptake assay.

6) Figure 5. What do the arrows indicate? GFP positive cells? Please indicate in figure legend.

7) Figure 6. Please describe what the different colors represent for microscopy images in each figure legend where appropriate.

8) There are a several typographical errors in the text that should be corrected.

Referee #3 (Comments on Novelty/Model System):

The work has strong medical relevance as atherosclerosis is a major medical concern in western societies.

The manuscript is written well and logically presented. The figures could be improved at certain points (see later).

The manuscript's main finding is that PGC1a is a key molecule in atheroprotection induced by CLA. The manuscript contains a full circle of in vitro cellular studies, animal models and human data. This is the main strength of the manuscript.

Referee #3 (Remarks):

The work has strong medical relevance as atherosclerosis is a major medical concern in western societies.

The manuscript is written well and logically presented. The figures could be improved at certain points (see later).

The manuscript's main finding is that PGC1a is a key molecule in atheroprotection induced by CLA. The manuscript contains a full circle of in vitro cellular studies, animal models and human data. This is the main strength of the manuscript.

The following minor points should be addressed before publication:

1. The manuscript is started with a figure derived from pathway analysis. The manuscript itself, and the data presented supports the suggestion of the molecular network and the hub position of PGC1a. Yet, I am not convinced that, as a general rule, a derivative type of figure, such as a network proposed by a pathway analysis, is a good way to start an experimental paper.
2. The authors should make sure that they use unique identifiers and the right format when referring to genes (e.g. Abca1 instead of ABCA-1, etc.).
3. It would be very useful to get an idea on the CT values of the investigated RT-qPCR reactions. In the format the data is presented, the relative changes can be seen easily, but we have no impression on the expression levels of these mRNAs.
4. Fig 3C in the present format (i.e. that is accessible to the reviewer) is barely readable. The inclusion of the drop-down menu-like databox might show us all the numerical data related to the experiment, but I highly doubt that this is the best way to show these data.
5. Although the general level of the writing is good, there are some remaining errors in the text that should be taken care of (e.g. page 7, "cluster induced regulated").

1st Revision - authors' response 05 May 2013

Referee #1 (Comments on Novelty/Model System):

I have struggled a bit when reviewing this paper. In general, the quality of the data aquisition and presentation including additional controls and statistics, which appears to be amenable to improvement. However, the authors should in addition perform some essential experiments in PGC-1a-deficient mice by cholesterol feeding or crossing them into an Apoe-/- background.
Should these experiments be provided with adequate results, the paper may become acceptable for publication.

Referee #1 (Remarks):

Belton et al.: Macrophage PPAR gamma Co-activator-1 alpha participates in repressing foam cell formation and atherosclerosis in response to conjugated linoleic acid.

The authors intended to further clarify the mechanism of CLA-induced AS regression in cholesterol-fed Apoe-/- mice. Their lab previously demonstrated reduced macrophage infiltration in lesions and reduced adhesion molecule, MMP-9 and pro-inflammatory cytokine expression by CLAs, while Ringseis et al. reported reduced lipid accumulation and enhanced efflux. By differential expression screening in aortic extracts from cholesterol- and +/- CLA-fed Apoe-/- mice, PGC-1a emerged as the hub of a candidate gene cluster. Subsequent experiments aimed at pinpointing the site of PGC-1a involvement into lesion macrophages and to relate it to human plaque samples.

It seems that the authors have somewhat made a second step before the first one: The PGC-1a-deficient mice are available and (on standard chow) atherosclerosis is obviously not a prominent feature of their phenotype. So why did they not first study cholesterol feeding in PGC-1a-/- mice or cross them with Apoe-/- mice (see Stein et al. 2010) before the bone marrow transplantation experiments? In these mice and their PG-1a +/+ controls the effect of CLAs and its mediation by PGC1a could have been tested directly in vivo.

We thank the reviewer for raising this important point. Indeed, on review of the literature, there are no studies to date which have investigated the effect of cholesterol feeding in PGC-1a$^{-/-}$ animals. We have now comprehensively addressed this and have included the novel data in the revised manuscript.

PGC-1a$^{-/-}$ and PGC-1$^{+/+}$ animals were randomised at 40 days of age to receive a 1% cholesterol chow for either 16 or 20 weeks. In addition to analysing atherosclerotic lesion formation, quantification of serum cholesterol and triglycerides in all animals was also performed. There was no change in serum cholesterol or triglycerides in PGC-1a$^{-/-}$ mice compared to their littermate controls following either 16 or 20 weeks high cholesterol feeding. Furthermore, comprehensive analysis of lesion formation using *en face* analysis showed that PGC-1a gene deletion does not impact on atherosclerotic lesion formation at either 16 or 20 weeks following high cholesterol feeding. Indeed there was no eivdence of any lesion formation in PGC-1a$^{-/-}$ mice. All novel data is now included in the revised manuscript as Supplementary Figure 9.

In addition, and as the reviewer has correctly indicated, Stein et al., have successfully generated the apoE$^{-/-}$/PGC-1$^{-/-}$ mouse. As reported in their publication (Stein et al., PLOS One 2010) global gene deletion of PGC-1a does not increase atherosclerotic lesion formation or plaque burden. However, in that study they provided evidence that deletion of PGC-1a increases CD68 macrophage and ICAM-1 positive cells in the atherosclerotic lesion. Therefore, due to the absence of a clear differential phenotype in both PGC-1a$^{-/-}$ and apoE$^{-/-}$/ PGC-1α models, it is not considered an efficient use of our animal models to examine the effect of CLAs and its mediation by PGC-1a directy in these murine models.

However, the previously published evidence for alteration in macrophage content in apoE$^{-/-}$/PGC-1a$^{-/-}$mice provides a strong rationale for our study to directly examine the role of macrophage PGC-1a$^{-/-}$ in the context of atherosclerosis.

Specific points:

p.5: Cyp7A1 initiates the classical (not the alternative) bile acid pathway.

We apologise for this error and have revised the statement accordingly on Page 5 of the revised manuscript.

p.7, design: The 1% CLA supplement was only contrasted to 'no supplement'. An additional control e.g. by an OA or LA supplement might have been of interest, although both fatty acids are not inert. LA shares the same caloric load, the same degree of unsaturation (even the same n-6, 12c-configuration with the t10,c12-CLA isomer) and covers direct PPARgamma effects.

Initially we used the 9,11 CLA isomer as a control in our *in vivo* experiments as we believed this isomer to be an attractive control in our regression model. However following en-face analysis of aortic lesions it was evident 9,11 CLA supplementation alone did not induce regression of atherosclerosis when compared to animals fed a diet supplemented with an isomeric CLA blend (98% versus 48% respectively). These results led us to conclude that the effects on regression of the following CLA blend supplementation were *not due to the PUFA nature of CLA*.
However, we agree with the reviewer's comments that linoleic acid could have been included as a more suitable control diet as performed in an elegant study by Arbon´es-Mainar et al. 2006 who demonstrated a reduction in atherosclerotic lesions in animals fed a 9,11 CLA supplemented diet when compared to a linoleic acid fed control diet.
However, in order to address the main aim of our study, which was to elucidate the genes mediating pathways/networks involved in the regression of atherosclerosis we selected the 1% cholesterol diet

as our control diet to compare with the CLA supplemented diet to generate a significantly stringent divergent dataset. This dataset allowed us significant bioinformatic power to elucidate clear pathways involved in regression of atherosclerosis, which we could fully relate to CLA blend supplementation. We appreciate this helpful suggestion by the reviewer and will include linoleic acid in future *in vivo* experiments. Indeed, we have now employed linoleic acid as a control in our *in vitro* studies and have recently published that isomeric blend of CLA isomers inhibited monocyte migration whereas linoleic acid treatment had no effect on migration (McClelland et al. Atherosclerosis 2010(1):96-102).

Please mention how the aortic RNA extracts used for differential expression analysis were standardized (by wet weight / total RNA content / a house-keeping gene / internal standard etc.)?

Aortic RNA was standardised using total RNA content (1µg of total RNA was used consistently for all reverse transcription reactions). In addition 18S was used as as a housekeeping gene for all transcripts analysed. We have included this on page 21 of the revised manuscript. In addition we have provided a Table detailing the CT values for each gene transcript analysed by real time PCR This Table is included as Supplementary Table 2 in the revised manuscript.

p. 8, top: How many of the n=10 endartierectomy patients were symptomatic or asymptomatic. Their potentially confounding medication (statins, fibrates, ASA, NSAIDs, antidiabetics, antioxidants, smoking habits) and lipid profiles must be given.

We apologise for not including this in our original submission and agree that this is important in the context of our clinical data. A table detaling patient details including disease status, age, cholesterol, LDL, HDL, triglycerides, diabeteic status, ASA, statin treatment and smoking status where applicable is now included as Supplementary Table 3, and referred to on page 8 of the revised manuscript.

p.8, bottom: Please mention, how the RNA extraction from plaques and the PCR was standardized to allow quantitative comparisons of asymptomatic vs. symptomatic plaques.

RNA from asymptomatic and symptomatic plaques was standardised using total RNA content (1µg of total RNA was used consistently for all reverse transcription reactions) in addition to using 18S as a housekeeping gene for all transcripts analysed. We have included this detail on page 8 of the revised manuscript.

Among the PGC-1a interacting transcription factors LXR is missing, which would be of special interest in the setting of regression of AS.

We have now completed analysis of LXRa expression in symptomatic and asymptomatic human plaques. As LXRa also regulates ABCA-1 we also included this in our analysis. Interestingly there is an inverse relationship between PGC-1a expression and LXRa expression in human atherosclerotic disease. Indeed previous studies have shown that LXRa in the liver down-regulates PGC-1α which is in contrast to the effects observed in white fat where LXRa has no effect on expression of PGC-1a. This suggests that the effects of LXR on PGC-1a are tissue-specific (Laffitte et al., 2003). We have included this novel data as part of Supplementary Figure 3 and have discussed the findings on page 9 of the revised manuscript.

p.9, bottom: The efflux (Fig. 3E) assay should be described in more detail as the various fatty acids influenced lipid uptake (Fig. 3, A-D): How was equal 3H-cholesterol labelling of cells assured? We have descirbed the assay in more detail as requested.

Cells were left for 48hrs after labelling to ensure complete uptake of [^{3}H] Cholesterol. Furthermore cells were treated in media containing 0.2%BSA for 24 hrs to equilibrate cells as previously described (Weibel et al. ATVB 2009) In addition, the % efflux due to passive diffusion was

determined and subtracted from values for those of the acceptors to definitively calculate efflux. We have clarified this on page 10 of the revised manuscript.

How was efflux espressed (% secreted of accumulated intracellular label / % of total 3H-cholesterol added for labelling)?

Cholesterol efflux was calculated as the percentage of radioactivity([^{3}H] -cholesterol) associated with medium over the sum of radioactivity of both medium and lysate. This is now included on page 10 of the revised manuscript.

As HDL and apo A1 were used as acceptors please show both data.

The percentage of cholesterol efflux to apoA-1 was approx <1.5%for each treatment which is very small and showed no significant difference between treatments. For this reason and to ensure clarity of the experiments and the data generated we have now just included the data for HDL in the revised manuscript.

The interpretation of absence or presence of significance in efflux data should be reconsidered (n=3, large SEMs, multiple t-testing ?).

We agree that this should be reconsidered in particular in relation to the inter experimental variation in efflux assays which resulted in a large SEM. Please find below the detailed raw data of this analysis. For this reason we have refined our interpretation of this on page 10 of the revised manuscript. If deemed appropriate by the reviewers this raw data can be included as part of the Supporting Information in the manuscript.

	**N=1**	**N=2**	**N=3**	**Average**	**Std Dev**	**Std Error**
DMSO	4.050118	7.168304	2.581366	4.599929	2.342375	1.352371
25uM 9,11 CLA	7.079185	9.593246	3.023678	6.56537	3.314787	1.913793
25uM 10,12 CLA	7.448987	9.507377	3.594731	6.850365	3.001434	1.732879
25uM Blend CLA	7.747307	8.799396	5.068296	7.205	1.923759	1.110683
10uM Trog	5.026082	6.591814	2.609861	4.742586	2.006057	1.158198
25uM OA	5.327129	4.990144	5.998997	5.438757	0.513607	0.296531
10uM LXR agonist	5.456728	3.554053	8.859581	5.956787	2.687881	1.551849

p.10 and Fig. 4: Why were RAW cells first oxLDL-loaded before exposure to CLAs? OxLDL loading provides a complex mixture of bioactive lipids and will per se stimulate PPARgamma and LXR, so effects of CLAs on PPARg and LXR might be obscured.

We apologise that the experimental strategy for the analysis of foam cell formation was not described adequately in our original submission. We have now clarified this. In all experiments RAW 264.7 macrophage cells were first pre-treated with 25uM of CLA isomers and controls for 24 hours and thereafter treated with oxLDL for four hours. This is now included on page 11 of the revised manuscript. The reason for this strategy is that as the reviewer correctly points out, oxLDL loading can stimulate PPARg and LXR which would confound the effects of CLA.

In addition to expression relative to DMSO control, which was set to 1 for each transcript, the relative expression to a common house keeping gene should be given to provide an estimate of the absolute expression levels (e.g. Cyp7b1 should be rather low in macrophages).

In our real time PCR analysis the housekeeping 18S is consistently used as a control. To determine the expression of all other genes the the CT values for 18S are subtracted from those of each target gene and then relative quantification to DMSO is performed. As a general principle the lower the

CT value the larger the amount of target expression. A full list of CT values for 18S and all target genes analysed in murine aorta, human plaque, RAW 264.7 macrophage cells and GFP- PGC-1α transfected RAW macrophage cells is provided in Supplementary Table 3 in the revised manuscript.

Fig. 4B: UCP-1 was stimulated by c9,711-CLA but not by the blend containing 90 c9,t11-CLA (in contrast to PGC-1a and Cyp7b1). This should be discussed.

We have discussed this on Page 11 of the revised manuscript. Induction of UCP-1 is sensitive to only one isomer, c9,t11CLA and not to the blend. The response to the blend suggests an antagonistic effect of the 10,12 CLA isomer in the CLA blend (80:20, c9,t11CLA:t10,c12CLA). This is supported by a previous study which showed isomeric differences of CLA on UCP-1. Metges et al., reported that *cis*-9,*trans*-11 CLA increased , whereas *trans*- 10,*cis*-12 CLA inhibited UCP1 mRNA expression in primary adipocytes *(Metges et al., CLA affect lipid metabolism differently in primary white and brown adipocytes of Djungarian hamsters, Lipids. 2003, 38: 133–1142)* .

p.11 bottom and Fig. 5A: Only two weakly PGC-1a-GFP expressing cells are shown in the small insert. Please provide an overview (like in Fig. 3 A) showing more PGC-1a-GFP+ and possibly simultaneously GFP-negative cells (like in the vector controls) to allow comparison of ox-LDL-accumulation in these cells.

Although transfection of the empty GFP and PGC-1α-GFP vectors was successful and allowed for the direct visualization of GFP proteins and the effect on foam cell formation the transfection efficiency was low. However, to provide a more comprehensive view of the effect of PGC-1α over expression on foam cell formation we have now included a timepass video of oxLDL flux in PGC-1α transfected and non-transfected RAW macrophages which illustrates inhibition of foam cell formation in PGC-1α over expressing cells over time.

p. 11 and Fig. 5C: As PGC-1a also activates LXR (Oberkofler et al.) and LXR induces ABCA1, their mRNA levels should also be analysed in PGC-1a over-expressing cells.

Whilst we appreciate that identification of gene regulation of LXR and ABCA-1 would have been interesting the low transfection efficiency precluded us from doing this. During the revision of this manuscript we revised our experimental approach to investigate the effect of silencing PGC-1α expression in the RAW264.7 mouse macrophages. RAW macrophages were transfected with Dharmacon Accell siRNA in Accell media without transfection reagent. Transfection with 2μM siRNA resulted in a 24% decrease in PGC-1α mRNA expression which increased to 53% with 5μM siRNA. Even though the decrease in PGC-1α expression was not complete, there were significant changes in expression of downstream target genes. There was a reduction in UCP-1 (51% with 5μM), Cyp7b1 (51% with 2μM), and a marked increase in the expression of the scavenger receptor CD36 (211% with 5μM) which suggests that decreased PGC-1α expression may result in increased uptake of oxLDL and again suggests an inverse relationship between PGC-1α and CD36. However, in the absence of complete knockdown of PGC-1α we did not detect any significant change in LXRalpha expression.

There are two possible explanations as to why nucleotide transfections to decrease and increase PGC-1α in the RAW264.7 macrophages were unsuccessful. Firstly, the macrophage is classified as a difficult to transfect cell type (Burke et al., 2002). In contrast to other cell types, macrophages have phagocytic functions which impedes efficient transfection as endocytosis of the siRNA or DNA complexes is likely to be digested by lysosome resident nucleases following endosome-lysosome fusion (Burke et al., 2002, Nagata et al., 1983). The second explanation relates directly to PGC-1α. The PGC-1α protein has a short half life of only 2.28hrs which increases to 6.27hrs upon p38MAPK mediated phosphorylation (Puigserver et al., 2001), has a high turn over rate and is targeted for ubiquitin mediated degradation (Puigserver et al., 2001, Olson et al., 2008, Sano et al., 2007).
Furthermore, we have performed Affymetrix gene array analysis of bone marrow derived macrophages from PGC-1-/- animals. Although the data from that study is part of manuscript currently under review elsewhere we can report that in the PGC-$1^{-/-}$ macrophages there was no change in LXRa and a small but non-significant change in Abca-1 expression

Discuss the dramatic reduction of foam cells in the light of the minor effect on CD36 and the increase in SRA-1.

Although the inhibition of foam cell formation is clearly visualised in PGC-1α over expressing cells, it is important to note that the effects of PGC-1α over expression on gene regualtion may be confounded as a result of low transfection efficiency of the RAW macrophage cells. We have discussed this on page 12 of the revised manuscript.

A measure of the absolute abundance of the various transcripts should be given.

A comprehensive list of CT values for all transcripts analysed in this manuscript are included in Supplementary Table 2 of the revised mauscript.

p. 11/12 and Fig. 6D: The % foam cells from wt mice somewhat arbitrarily set to 100%, resulting in '140 %'(?) in PGC1-a-KO? Rather use the same quantification as in PG1a-OE (Fig. 5B).

We apologise if we did not clarify the quanitification of foam cell formation. The strategy used for quantification in both Fig 6D and Fig 5B was the same, where foam cell formation was set to 100% for the control either WT in the case of Fig 5B or DMSO treated BMDMs for Fig. 6D

Fig. 6E: The technical quality of the micrographs is insufficient to allow comparison the effects.

We apologise for the poor tehnical quality of the images in our original submission. We have now changed Figure 6E to provide high resolution images of BMDMs from PGC-1a$^{-/-}$ mice. As can be clearly seen from the images (Figure 6E) and the graph (Figure 6F) there is no difference in foam cell formation between any of the treatment groups.

p.12 bottom: Why were CLA supplements not tested in BM-reconstituted LDLR-/- mice?

We agree that this is an excellent suggestion for our future experiments. Although it is challenging to investigate the effect of CLA on regression in this model, over the next 12 months we intend to expand on the data presented in this manuscript and address the effect of CLA in mice lacking macrophage PGC-1a. However, as we are the first group to show that macrophage PGC-1a plays a role in atheroprotection the authors concur that investigating the effect of CLA in macrophage PGC-1a$^{-/-}$ mice is currently beyond the scope of this manuscript.

Discussion:
p. 14, top: ...CLA inhibits both CD36 and SRA-1... but PCG-1a-GFP transfection did not (Fig. 5 C). This should be discussed.

Although PGC-1a overexpression inhibited CD36 expression the magnitude was indeed less than that observed with CLA treatment of RAW macrophages. This is likely due to low transfection efficiency. We have discussed this on Page 12 of the revised manuscript.

p.16: Björkem et al. established Cyp27, not Cyp71b, as a major player in macrophage cholesterol handling.

We apologise for this error and have revised accordngly on Page 16 of the revised manuscript

p. 21: Statistics: t-test is not adequate for data shown in Fig.3 A to G and Fig. 4A-D.

We appreciate the input and comment on our statistical analysis. We have now repeated the statisical analysis for the experiments described in Figures 3 and 4 using ANOVA.

All the information of the two-column panels of Fig. 1B-C (with control set to 1) can be presented on much less space.

We have revised this figure accordingly and included all data from the analysis as a single graph. This is inlucded as part of Figure 1 in the revised manuscript.

All n = x, now mentioned in methods p.18 ff., should better be given in legends to figures.

This has now been changed as requested.

_

Referee #2 (Remarks):

Summary: Paper follows up on previous observations that conjugated linoleic acid (CLA) regresses atherosclerotic plaque in cholesterol-fed mice. Transcriptional analysis of atherosclerotic plaque from CLA-fed mice implicates PGC1alpha as a candidate mediating the effect of CLA on regression. Human atherosclerotic plaque was shown to have increased expression of PGC1alpha. Studies in mice showed that overexpression of PGC1alpha was associated with reduced foam cell formation in an in vitro assay while knockout was associated with an increase. Macrophage specific deletion of PGC1a in an athero-susceptible mouse model was associated with increased aortic plaque area. The authors conclude that PGC1alpha plays a role in atheroprotection.

Comments:

1) The manuscript identifies PGC1alpha as a potential driver of the anti-atherosclerotic effect of CLA. While evidence is presented that demonstrates that lack of PGC1alpha influences foam cell formation and atherosclerosis a key experiment is missing showing that CLA has no effect on foam cell formation or progression/regression of atherosclerosis in PGC1alpha knockout mice.

The overall aim of the manuscript was to elucidate novel pathways and identify potential "hub" genes, which govern the regression of atherosclerosis *in vivo*. In our study CLA was employed as an interventional tool to identify such genes and then to define a functional role of the hub genes in the context of atherosclerosis. To this end, the focus of the manuscript is investigating an atheroprotective role for PGC-1ain the macrophage cell, which we have achieved through loss and gain of function experiments *in vitro* and *in vivo* respectively. However, the reviewer raises an interesting point.

To date there has been no published study which investigated the effect of cholesterol feeding in PGC-1a$^{-/-}$ animals. As detailed above in response to reviewer 1, during the revision of this manuscript we comprehensively addressed this. We are delighted to include the data in the revised manuscript. PGC-1a$^{-/-}$ animals were randomised at 40 days of age to receive a 1% cholesterol chow for 16 or 20 weeks. As presented in the revised manuscript there was no change in serum cholesterol or triglycerides in PGC-1a$^{-/-}$ mice compared to their littermate controls at either 16 or 20 weeks. Furthermore, comprehensive anaysis of lesion formation use *en face* analysis showed that PGC-1a gene deletion does not impact on atherosclerotic lesion formation at either 16 weeks or 20 weeks. Indeed there was no eivdence of lesion formation in PGC-1a$^{-/-}$ knockouts. All novel data is included in the revised manuscript as Supplementary Figure 9. In addition, Stein et al., have successfully generated the apoE$^{-/-}$/PGC-1$^{-/-}$. As reported in their publication in (PLOS One, 2010), global gene deletion of PGC-1a does not increase atheroscerotic lesion formation or plaque burden.

Therefore, due to the abscence of a clear differenital phenotype in both PGC-1a$^{-/-}$ and apoE/ PGC-1a$^{-/-}$ modles fed a high cholesterol diet it is not considered an efficient or ethical use of our animal models to examine the effect of CLAs and its mediation by PGC-1a directy in these models.

Ideally this would be done by measuring atherosclerosis development in Ldlr knockout mice (or apoE knockout) with bone marrow transplant with cells from Pgc1alpha knockout mice with and without 1% CLA diet. If the hypothesis is correct there should be no effect of CLA on atherosclerosis in these mice.

Although it is challenging to investigate the effect of CLA on regression in this model, over the next 12 months we intend to expand on the data presented in this manuscript and address the effect of

CLA in mice lacking macrophage PGC-1a. However, as we are the first to show that macrophage PGC-1a plays a role in atheroprotection the authors concur that investigating the effect of CLA in macrophage in bone marrow transplant PGC-1a$^{-/-}$ mice is currently beyond the scope of this manuscript.

2) The uptake of oxidized LDL in BMDMs (figure 6E) appears to be much less that what was seen using RAW cells (figure 2A). Are there any differences in oxidized LDL uptake in BMDMs from wild type of apoE knockout mice incubated with and without CLA? This should be tested.

This is an important comment and we have now examined uptake of OxLDL in WT apoE mice. The data is now provided in Supplementary Figure 7 in the revised manuscript . The reviewer is correct in notin that there are differences in oxLDL uptake between cell lines such as RAW macrophages and THP-1 macrophages and primary cell culture models such as thioglycollate elicited macrophages and bone marrow derived macrophages. We have also performed experiments in peripheral blood monocyte derived macrophages where we also observe differences in the uptake of oxLDL.

3) The data in supplemental figure 3A showing PGC1alpha expression in human plaque is useful in demonstrating reduced PGC1alpha expression in plaque versus relative disease-free tissue. However, the comparison showing expression of PGC1alpha in plaque from symptomatic versus asymptomatic patients is dependent on the assumption that the plaque burden in symptomatic patients is greater than that in asymptomatic patients which may or may not be the case. Please delete figure 2C and replace with supplemental figure 3.

We have moved Supplementary Figure 3 to the main body of the text. In addition we have now further supported this figure with real time PCR analysis of PGC-1a expression in asymptomatic and symptomatic patinets. In addition, the clinical classification of disease status, cholesterol levels, diabeteic status, statin and aspirin use for all patients recruited in the study is now provided as Table 1 in the Supporting Information.

4)Figure 3E. The cholesterol efflux assay was done using HDL and apoA-I as acceptors. Which is being shown in this figure?

Efflux to HDL is represented in Fig 3E. This has been clarified on page 10 of the revised manuscript.

Efflux to both HDL and apoA-I should be reported.

In our original submission we stated that efflux to both HDL and apoA-1 had been performed. However, the percentage of cholesterol efflux to apoA-1 was approximately <1.5%for each treatment which is relatively low and no significant changes were observed. Therefore, for the purpose of clarity in the manuscript we have now removed the references to quantification of efflux to apoA-1.

There appears to be increased efflux for cells treated with c-9,t-11 CLA and t-10,c-12. The lack of significance of seems to be due more to experimental variability than anything else.

This is indeed correct. The raw data for these experiments is provided as a Table in response to Reveiwer 1. As is evident there was a degree of inter experimental variation resulting in large SEM. In light of this, we have revised out interpretation of this data. We would be happy to provide this raw data in supporting information should it be deemed appropriate

Increased efflux would be consistent with the increase in Abca1 shown in figure 1B and may be related to the increase in Cyp7b1 since this would be a mechanism for hydroxylated sterol removal. Please comment in the Discussion on the possibility that increased efflux contributes the reduced foam cell formation seen in response to CLA.

This is an interesting point and we have discussed this on page 16 of the revised manuscript.

5) Page 9. Cholesterol uptake assay is actually an acetylated LDL uptake assay using a tritiated cholesterol label. Please change to cholesterol uptake assay to acetylated LDL uptake assay

We apologise for this inaccuracy. This has been corrected on page 9 of the revised manuscript.

6) Figure 5. What do the arrows indicate? GFP positive cells?

The arrows depicting the GFP positive cells have been explained in the legend for Figure 5 on page 36 of the revised manuscript.

7) Figure 6. Please describe what the different colors represent for microscopy images in each figure legend where appropriate.

This has been included in each figure legend where micropscopy images are described.

8) There are a several typographical errors in the text that should be corrected.

We apologse for these errors and have corrected all typographical errors

---

Referee #3 (Comments on Novelty/Model System):

The work has strong medical relevance as atherosclerosis is a major medical concern in western societies.
The manuscript is written well and logically presented. The figures could be improved at certain points (see later).
The manuscript's main finding is that PGC1a is a key molecule in atheroprotection induced by CLA. The manuscript contains a full circle of in vitro cellular studies, animal models and human data. This is the main strength of the manuscript.

We sincerely thank the reviewer for the positive feedback and comments on our manuscript

Referee #3 (Remarks):

The work has strong medical relevance as atherosclerosis is a major medical concern in western societies.

The manuscript is written well and logically presented. The figures could be improved at certain points (see later).

The manuscript's main finding is that PGC1a is a key molecule in atheroprotection induced by CLA. The manuscript contains a full circle of in vitro cellular studies, animal models and human data. This is the main strength of the manuscript.

The following minor points should be addressed before publication:

1. The manuscript is started with a figure derived from pathway analysis. The manuscript itself, and the data presented supports the suggestion of the molecular network and the hub position of PGC1a. Yet, I am not convinced that, as a general rule, a derivative type of figure, such as a network proposed by a pathway analysis, is a good way to start an experimental paper.

We agree that considering the data presented in this manuscript a descritpive pathway analysis is not an ideal way to start this experimental paper. Therefore, we have removed Figure 1A from the main bosy of the text and moved it to Supporting Information Figire 1A.

2. The authors should make sure that they use unique identifiers and the right format when referring to genes (e.g. Abca1 instead of ABCA-1, etc.).

We apologise that the gene identifers were not formatted correctly and have ensured that these have been changed throughout the revised manuscript.

3. It would be very useful to get an idea on the CT values of the investigated RT-qPCR reactions. In the format the data is presented, the relative changes can be seen easily, but we have no impression on the expression levels of these mRNAs.

A complete list of CT values for 18S and all gene transcripts analysed in murine aorta, human atherosclerotic plaque, RAW macrophage cells and GFP- PGC-1a transfected RAW macrophage cells are now provided as a Table 2 in the Supporting Information. We agree that this reflects the expression levels of the transcripts in our analysis.

4. Fig 3C in the present format (i.e. that is accessible to the reviewer) is barely readable. The inclusion of the drop-down menu-like databox might show us all the numerical data related to the experiment, but I highly doubt that this is the best way to show these data.

We agree that this is not readable and therefore have revised the figure accordingly. We have removed the drop down box and provided the counts from the flow cytometry experiments as a separate panel.

5. Although the general level of the writing is good, there are some remaining errors in the text that should be taken care of (e.g. page 7, "cluster induced regulated").

We have corrected the typographical errors and apologise that they were present in our original submission.

2nd Editorial Decision 17 June 2013

Thank you for the submission of your revised manuscript to EMBO Molecular Medicine. We have now received the enclosed reports from the Reviewers that were asked to re-assess it. As you will see, while Reviewers 1 and 2 are now supportive, Reviewer 3 would like you to provide experiments with a cell-type specific knock-out to validate your conclusions further.

After additional discussion, including with Reviewers 1 and 2 through our cross-commenting procedure, I have decided to overrule Reviewer 3's request and provisionally accept your manuscript for publication, pending the following final amendments:

1) Reviewer 2 notes that the data in supplemental Figure 7 should be included in the manuscript.

2) The point raised by Reviewer 3 is nevertheless a valid one and should be mentioned in the Discussion as a limitation of your study.

3) As per our Author Guidelines, the description of all reported data that includes statistical testing must state the name of the statistical test used to generate error bars and P values, the number (n) of independent experiments underlying each data point (not replicate measures of one sample), and the actual P value for each test (not merely 'significant' or 'P < 0.05').

4) Please combine figure 1, 3 and 6 sub-panels into single figure panels (this can be easily achieved by reducing their size; but please make sure the resolution remains high). As for Fig. 2, it would be probably best if sub-panels a-c were combined into a new Fig.2 and sub-panel D were renumbered as Fig.3. This will imply the renumbering of a number of figures, and thus modifications in the text and figure legends. Please do so with the utmost care to avoid further to and fro of your manuscript!

Please highlight the changes in the main text with red lettering.

Your revised manuscript should be submitted within two weeks. Needless to say, the sooner we receive it the sooner I will be able to formally accept your manuscript.

***** Reviewer's comments *****

Referee #1 (Remarks):

The manuscript has been suitably revised and is now acceptable for publication.

Referee #2 (Remarks):

The authors have addressed all of my comments from the previous review. I was unable to find the data comparing OxLDL uptake in wild type and apoE KO mice +/- CLA that was mentioned as being in supplemental figure 7. This should be included the final version of the manuscript.

Referee #3 (Comments on Novelty/Model System):

The new data presented and the response to the concerns of the other two referees suggest to me that the model used might be prone to artifact and/or the experiments are not conclusive. An independent genetic model of macrophage depletion of PGC1 would be needed.

Referee #3 (Remarks):

The authors adequately responded to my concerns. However, the new data on PGC1 ko animals and the response to the other two reviewers comments regarding the athero phenotype of PGC1 KO animals on WT or ApoEKO background raises a flag.

In order to claim that macrophages PGC1a is implicated in atherosclerosis or in the protection against it, an independent genetic model would be needed. If it is not provided my worry is that macrophage PGC1a will be implicated in atherosclerosis based on BMT experiments only and without independent genetic verification either in the full knock out or in a cell type specific KO. This is likely to cause more confusion.

2nd Revision - authors' response 27 July 2013

We are delighted that you have provisionally accepted out manuscript “Evidence for the role of PGC-1alpha in mediating the anti-atherogenic effects of conjugated linoleic acid *via* inhibition of foam cell formation” for publication in EMBO Molecular Medicine.

We gratefully acknowledge and appreciate the valid and constructive comments rasied by the reviewers during the revision of our manuscript and appreciate their support of our manuscript Further to your correspondence on June 17th 2013 please find below our comments and revisions to this manuscript as requested by you.

1) Reviewer 2 notes that the data in supplemental Figure 7 should be included in the manuscript.

This figure has been removed from Supplementary Information and is now Figure 7F in the main

body of the revised manuscript.

2) The point raised by Reviewer 3 is nevertheless a valid one and should be mentioned in the Discussion as a limitation of your study.

We appreciate the concern of this reviewer and have provided a comment on this as a limitation of our study in the discussion (Page 19 paragraph 1) of the revised manuscript.

3) As per our Author Guidelines, the description of all reported data that includes statistical testing must state the name of the statistical test used to generate error bars and P values, the number (n) of independent experiments underlying each data point (not replicate measures of one sample), and the actual P value for each test (not merely 'significant' or 'P < 0.05').

In line with the Author Guidelines we can confirm that all required information relating to Statistical analysis and the actual p values have now been included for all figures in the main manuscript and in the supplementary information of the revised manuscript.

4) Please combine figure 1, 3 and 6 sub-panels into single figure panels (this can be easily achieved by reducing their size; but please make sure the resolution remains high).

We have combined the Figure 1, Figure 3 (labeled Figure 4 in the revised manuscript) and Figure 6 (labeled Figure 7 in the revised manuscript) into a single figure panels as requested.

As for Fig. 2, it would be probably best if sub-panels a-c were combined into a new Fig.2 and sub-panel D were renumbered as Fig.3.

Figure 2D is now a new Figure 3 in the revised manuscript.

This will imply the renumbering of a number of figures, and thus modifications in the text and figure legends. Please do so with the utmost care to avoid further to and fro of your manuscript!

We have carefully reviewed the changes to the figures and figure legends in the revised manuscript and have highlighted all changes in red.

Please highlight the changes in the main text with red lettering.

All changes have been highlighted in red as requested.
On behalf of the authors I wish to thank you for this opportunity to publish our work in EMBO Molecular Medicine.
