# Supplementary material for: Macrophage PPAR gamma Co-activator-1 alpha participates in repressing foam cell formation and atherosclerosis in response to conjugated linoleic acid
Source: EMBO Mol Med. 2013 Aug 21;5(9):1443–57. doi: 10.1002/emmm.201302587 (PMC3799497; doi:10.1002/emmm.201302587)
Supplement: Supplementary file 2 [file emmm0005-1443-SD2.pdf]

Supporting InformationTable of Contents

Supplementary Figures and Tables

Figure 1: Effect of CLA on regression of pre-established atherosclerosis in apoE$^{-/-}$ mouse 3

Figure 2: PGC-1α expression in murine aorta 4

Table 1: Patient clinical data 6

Figure 3: PGC-1α target gene expression in human atherosclerosis 7

Table 2: Real time PCR CT values 8

Figure 4: Effect of CLA isomers on adiopophilin mRNA expression 9

Table 3: Cholesterol uptake in RAW 264.7 macrophages 10

Figure 5: Quantification of cholesterol and triglycierides in CLA-induced regression 11

Figure 6: Measurement of intracellular Reactive Oxygen Species 12

Figure 7: Western blot of PGC-1α in BMDMs 12

Figure 8: PGC-1α protein and mRNA expression in peritoneal macrophage-derived foam cells and aorta from LDLR$^{-/-}$ mouse 13

Figure 9: (A) Serum cholesterol and triglyceride levels and (B) En face analysis of aorta from PGC-1α$^{+/+}$ or PGC-1α$^{-/-}$ mice on a high cholesterol diet 14

Figure 10: CLA increases AMPK phoshorylation *in vivo* 16

Video 1-3: Legends for Supplementary Video Files 1-3 17

Supplementary Methods

Immunohistochemical and Western Blot Analysis of Murine Aorta and Human Atherosclerotic Plaque 19

Quantification of PGC-1α expression in human atherosclerotic plaque 19

Supplementary Figure 11. Algorithm used in quantification of PGC-1α expression 20

Cell Culture 21

Primer sequences for real time PCR analysis and PGC-1a Genotyping 22

Flow cytometry 22

Cholesterol Uptake Assay 23

Cholesterol efflux Assay 23

Plasma cholesterol, triglyceride and lipoprotein analysis 24

Figure 1

A.

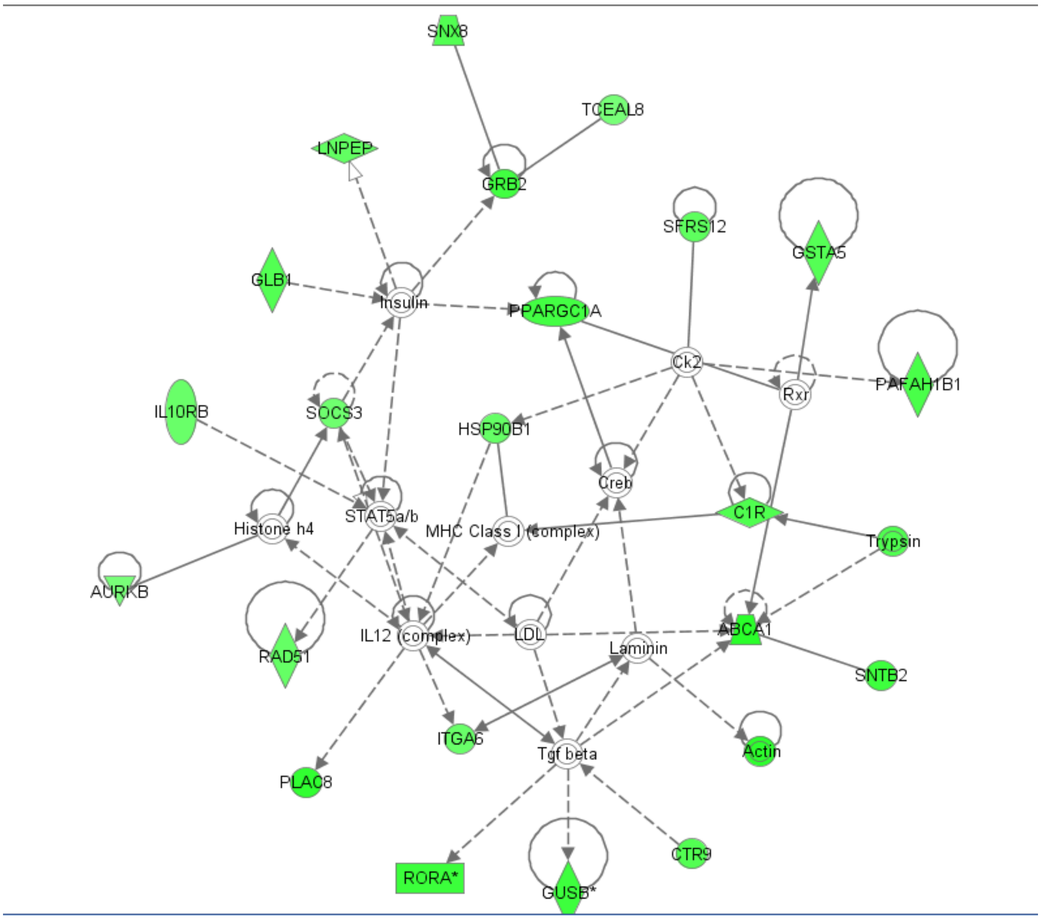

SNX8
TCEAL8
LNPEP
GRB2
SFRS12
GSTA5
GLB1
Insulin
PPARGC1A
Ck2
Rxr
PAFAH1B1
IL10RB
SOCS3
HSP90B1
Creb
C1R
Trypsin
Histone h4
STAT5a/b
MHC Class I (complex)
AURKB
RAD51
IL12 (complex)
LDL
Laminin
ABCA1
SNTB2
ITGA6
Tgf beta
Actin
PLAC8
CTR9
RORA*
GUSB*

B.

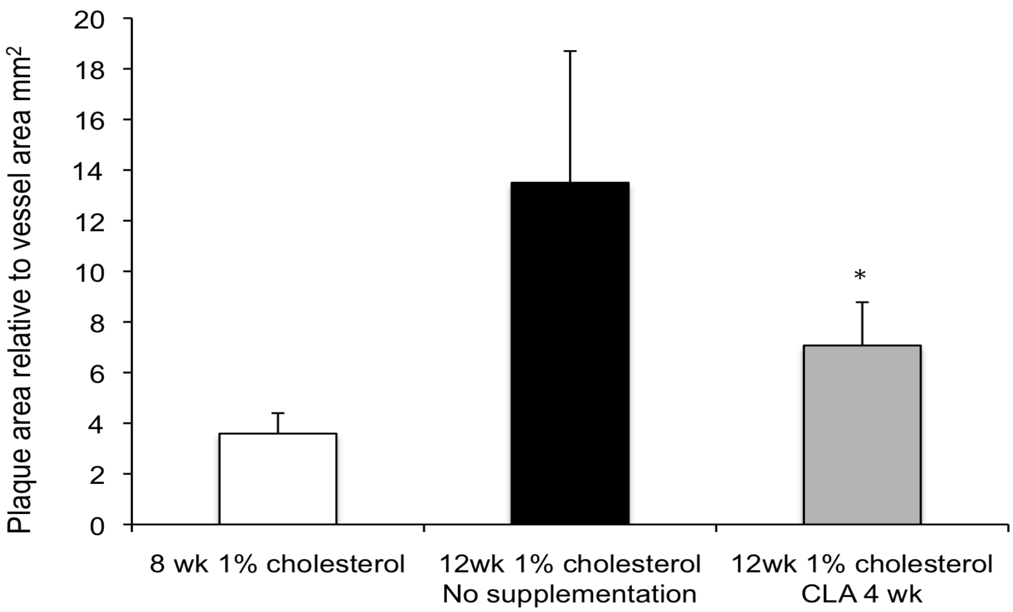

Plaque area relative to vessel area mm²
20
18
16
14
12
10
8
6
4
2
0
*
8 wk 1% cholesterol
12wk 1% cholesterol No supplementation
12wk 1% cholesterol CLA 4 wk

Supplementary Figure 1. Effect of CLA on regression of pre-established atherosclerosis in apoE$^{-/-}$ mouse

(A) Transcriptomic analysis of murine aorta in CLA-induced regression of atherosclerosis. PGC1-α was identified as a central hub gene located in the network most represented by genes significantly increased (shown in green) with CLA supplementation *in vivo.* (B) Plaque area relative to total aortic area in apoE$^{-/-}$ animals fed 1% cholesterol diet for 8 weeks (n=10) (open bars) or 12 weeks (closed bars) and those fed a 1% cholesterol diet for 12 weeks where the final 4 weeks was supplemented with 1% CLA blend (80:20, c-9,t-11: t-10,c-12 CLA) (n=10). Data are mean+/- SEM. Statistical analyses were performed using *t*-test, *P*=0.038 vs. apoE$^{-/-}$ 1% cholesterol diet 12wks.

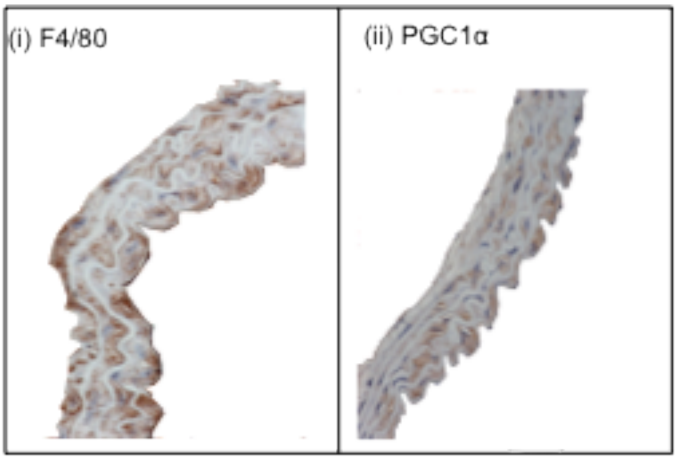

Supplementary Figure 2. PGC-1α expression in aorta of apoE$^{-/-}$ fed a CLA supplemented 1% cholesterol chow for 12 weeks (right panel). PGC-1α was localised to macrophage cells as confirmed by F4/80 staining of aortic tissue (left panel).

Table 1. Patient Characteristics

GENERAL			LIPID PROFILE				STATINS	DIABETIC STATUS		ANTI-PLATELET THERAPY	SMOKING STATUS		
Symptomatic Status	Age	Sex	CHOL (mmol/L)	LDL (mmol/L)	HDL (mmol/L)	Triglycerides	statins/dose	Diabetes	fibrates/dose	Aspirin/dose	smoking status	cigarettes/ day	duration of cessation
Asymptomatic	60	F	3.8	Unavailable	Unavailable	0.88	Atorvastatin (lipitor) / 10mg OD	no	none	Aspirin/75mg OD	ex	20-30	missing
Asymptomatic	71	M	5	1.14	3.57	0.64	Rosuvastatin (crestor) / 10mg OD	no	none	None	None	N/A	N/A
Asymptomatic	49	M	2.9	1.68	0.57	1.42	Atorvastatin (lipitor) / 40mg OD	no	none	Aspirin/75mg OD	ex	30	2 months
Asymptomatic	72	M	4.8	2.77	1.07	2.12	None	no	none	Aspirin/75mg OD	ex	unavialable	30 years
Asymptomatic	66	M	4	1.98	1.34	1.49	Rosuvastatin (crestor) / 10mg OD	no	none	Aspirin/75mg OD	ex	unavailable	30 years
Symptomatic	75	M	5.7	Unavailable	Unavailable	Unavailable	Atorvastatin (lipitor) / 80mg OD	no	none	Aspirin/75mg OD	None	NA	NA
Symptomatic	70	M	2.7	0.99	1.06	1.42	Atorvastatin (lipitor) / 80mg OD	no	none	Aspirin/75mg OD	ex	unavailable	unavailable
Symptomatic	63	M	4.4	2.44	1.67	0.06	Atorvastatin (lipitor) / 40mg OD	no	none	Aspirin/75mg OD	ex	15-20	5 weeks
Symptomatic	78	F	5.4	2.52	2.71	0.38	Atorvastatin (lipitor) / 20mg OD	no	none	Aspirin/75mg OD	current	5	NA
Symptomatic	83	M	3.7	Unavailable	Unavailable	Unavailable	Atorvastatin (lipitor) / 10mg OD	no	none	None	current	20	N?A

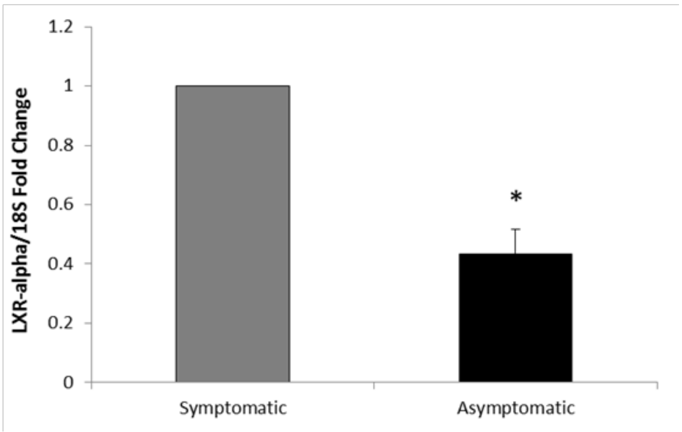

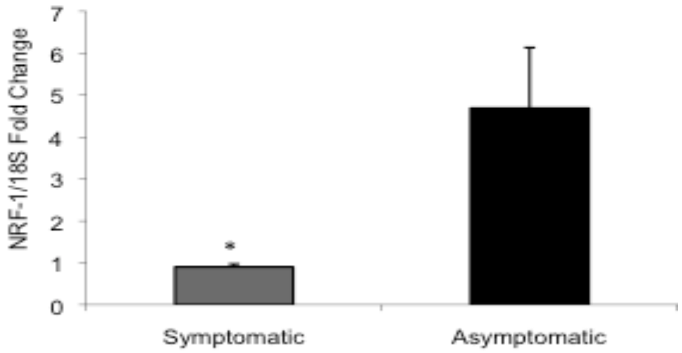

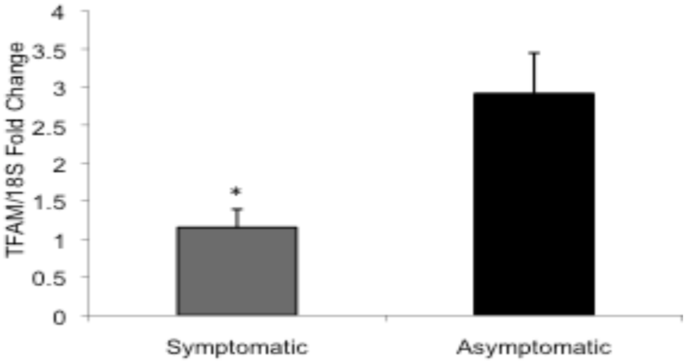

Supplementary Figure 3. PGC-1 α target gene expression in human atherosclerosis. Real time PCR analysis of LXRα, NRF-1 and TFAM mRNA expression in symptomatic and asymptomatic plaques. Data are mean+/- SEM of five independent experiments. Statistical analyses were performed using *t*-test where. Significant *P* values are as follows: *P*=0.033 for LXRα versus symptomatic and *P*=0.021 and 0.033 for NRF-1 and TFAM, respectively, versus asymptomatic plaques.

Supplementary Table 2: Real Time PCR analysis

Gene	CT Value Range
**RAW 264.7 Macrophage Cells**	
18S Control	10-11
Cyp7b1	24-26
PPARG	23-25
PGC-1a	17-19
UCP-1	32-34
SRA1	18-20
CD36	22-26
**PGC-1alpha GFP transfected RAW Macrophages**	
18S	10-11
PGC-1α	26-28
SRA-1	17-19
UCP-1	24-29
Cyp7b1,	26-28
PPARγ1	30-32
CD36,	21-23
**Murine Aorta**	
18S	9-10
PGC-1α	23-29
RORα	23-25
Abca1	21-23
Cyp7b1	27-29
UCP1	16-18
PPARγ	22-24
**Human Atherosclerotic Plaque**	
18S	10-12
PGC-1α	31-35
TFAM	24-28
NRF-1	26-29
LXR-α	21-27

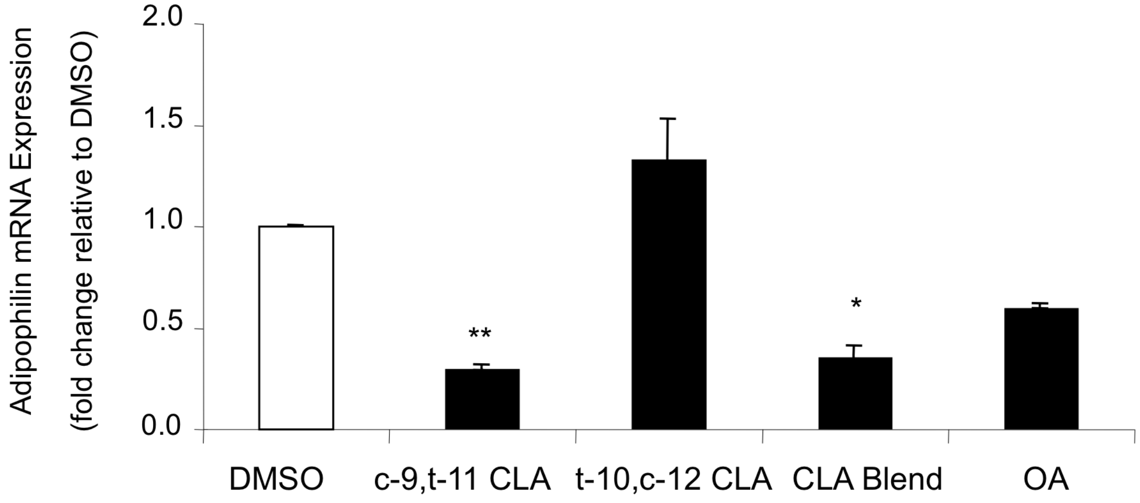

Supplementary Figure 4. Effect of CLA isomers on adiopophilin mRNA expression.

RAW 264.7 macrophage were pre-treated with (i) DMSO (ii) c-9,t-11-CLA (25 μM) (iii) t-10,c-12-CLA(25 μM) (iv) CLA blend (80:20 c-9,t-11-CLA: t-10,c-12-CLA) (25 μM) and (v) OA (25 μM) for 24 hrs prior to ox-LDL treatment (50 ug/ml) for a further 4 hrs.

Real time PCR analysis of Adipophilin mRNA expression, in oxLDL loaded cells following pre-treatment with DMSO, CLA isomers, CLA blend or control OA. Data are mean +/- SEM of 3 independent experiments. Statistical analyses were performed by ANOVA. Significant *P* values from left to right are *P*=0.0091 and 0.017 versus DMSO control.

Supplementary Table 3: Cholesterol uptake in Raw 264.7 Macrophage cells.

**Treatment**	**CPMA**	**P value versus DMSO**
**DMSO**	19824.69 +/-501.21	
**9,11 CLA**	17474.36+/-526.77	*P*=0.0091
**10,12 CLA**	17662.81+/-388.67	*P*=0.0082
**CLA Blend**	18648.75+/-304.1	*P*=0.0088

Cholesterol uptake in Raw 264.7 cells treated with h 25µM c9,t11-CLA, t10,c12-CLA, CLA blend (80:20 c-9t,11-CLA: and t10,c12-CLA), or DMSO for 24hours prior to addition 1µg/ml acLDL) and 1uCi/ml triatiated cholesterol for 4 hours. Scintillation counts were used to measure cholesterol content in cells. Data are mean +/- SEM of 3 independent experiments. Statistical analyses were performed using *t*-test.

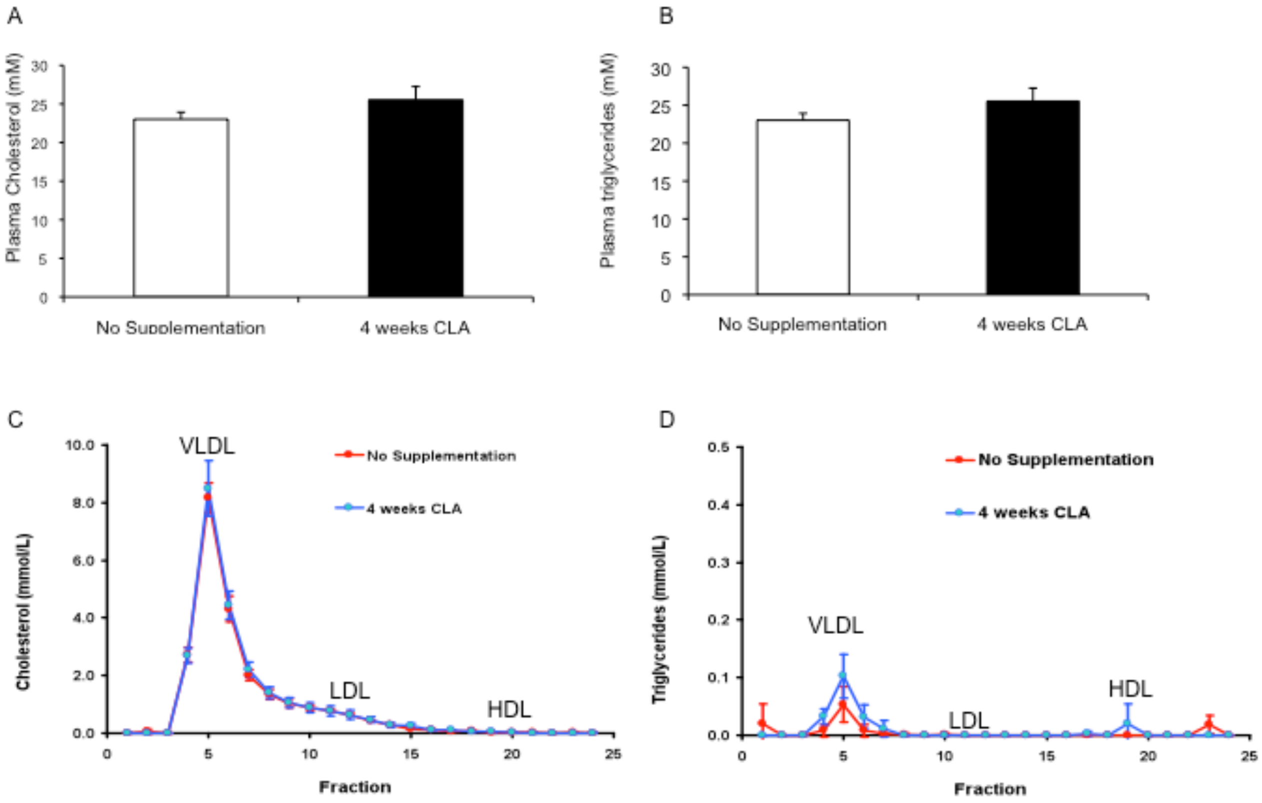

Supplementary Figure 5. Quantification of plasma cholesterol and triglycierides in CLA-induced regression *in vivo*. (A) Plasma cholesterol, (B) triglycerides and (C-D) mean FPLC lipoprotein profile analysis from apoE$^{-/-}$ mice fed a 1% cholesterol diet for 12 weeks with/without CLA supplementation for the final four weeks (n=3). All values are depicted as mean ± SEM.

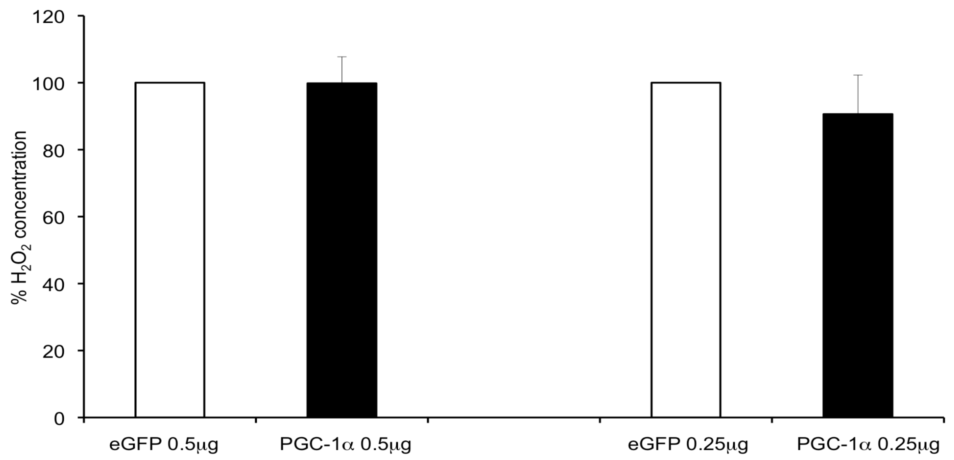

Supplementary Figure 6. Measurement of intracellular Reactive Oxygen Species. Percentage H_2O_2 concentration in RAW 264.7 macrophage cells transfected with 0.25µg or 0.5µg PGC-1α –GFP or eGFP. Data are mean +/- SEM and are representative of 5 independent experiments.

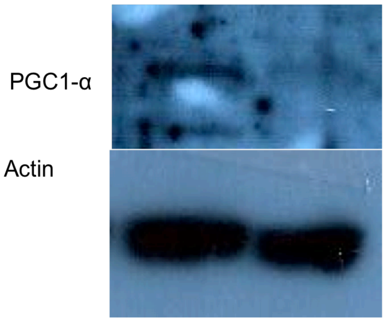

Supplementary Figure 7. Western blot analysis of PGC-1α expression in BMDMs from PGC-1α WT and KO animals.

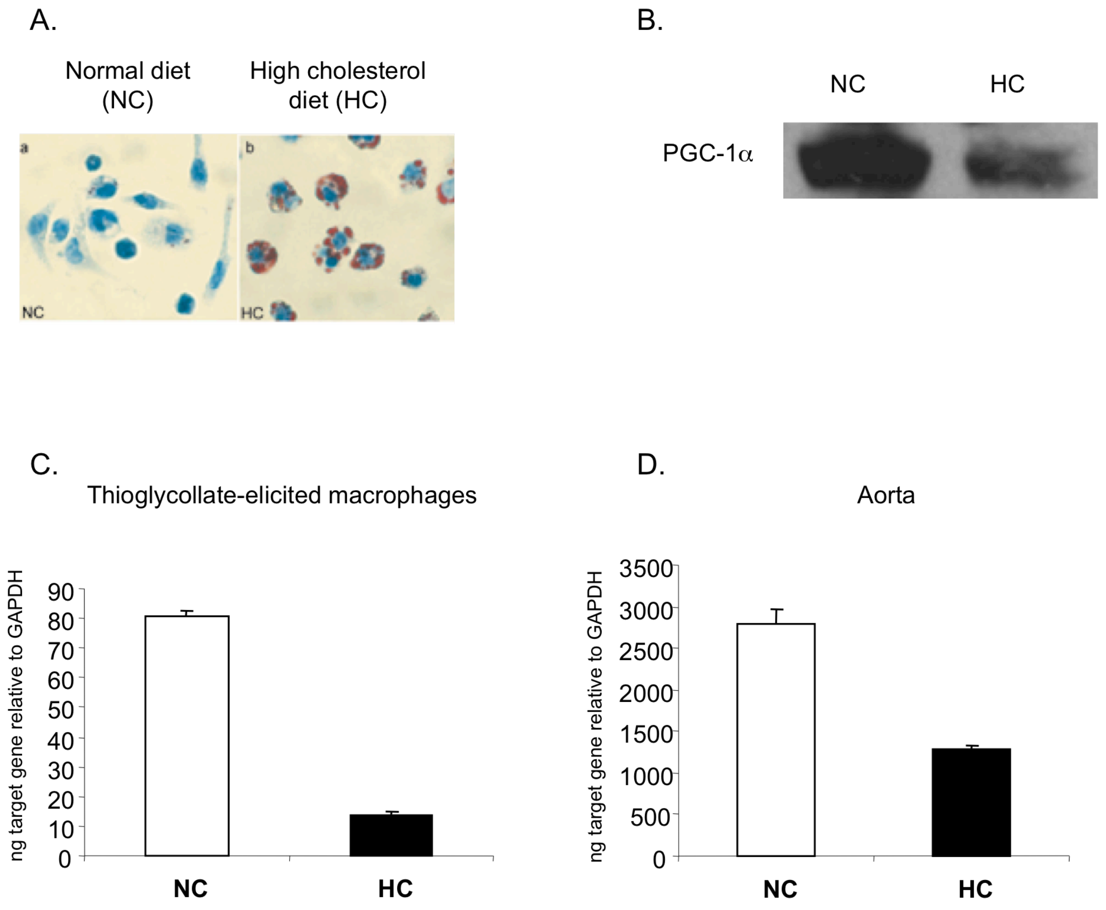

Supplementary Figure 8. PGC-1α protein and mRNA expression in peritoneal macrophage-derived foam cells and aorta from LDLR$^{-/-}$ mouse. (A) Oil Red O staining of macrophages from LDLR$^{-/-}$ male mice fed a normal chow (NC) or a high cholesterol diet for 4 months (HC). (B) PGC-1α protein levels measured by Western Blot and (C) PGC-1α mRNA expression in thioglycollate-elicited macrophages from LDLR$^{-/-}$ mice fed a NC and HC mice. (D) PGC-1α mRNA expression in aorta from LDLR$^{-/-}$ mice fed a NC and HC diet.

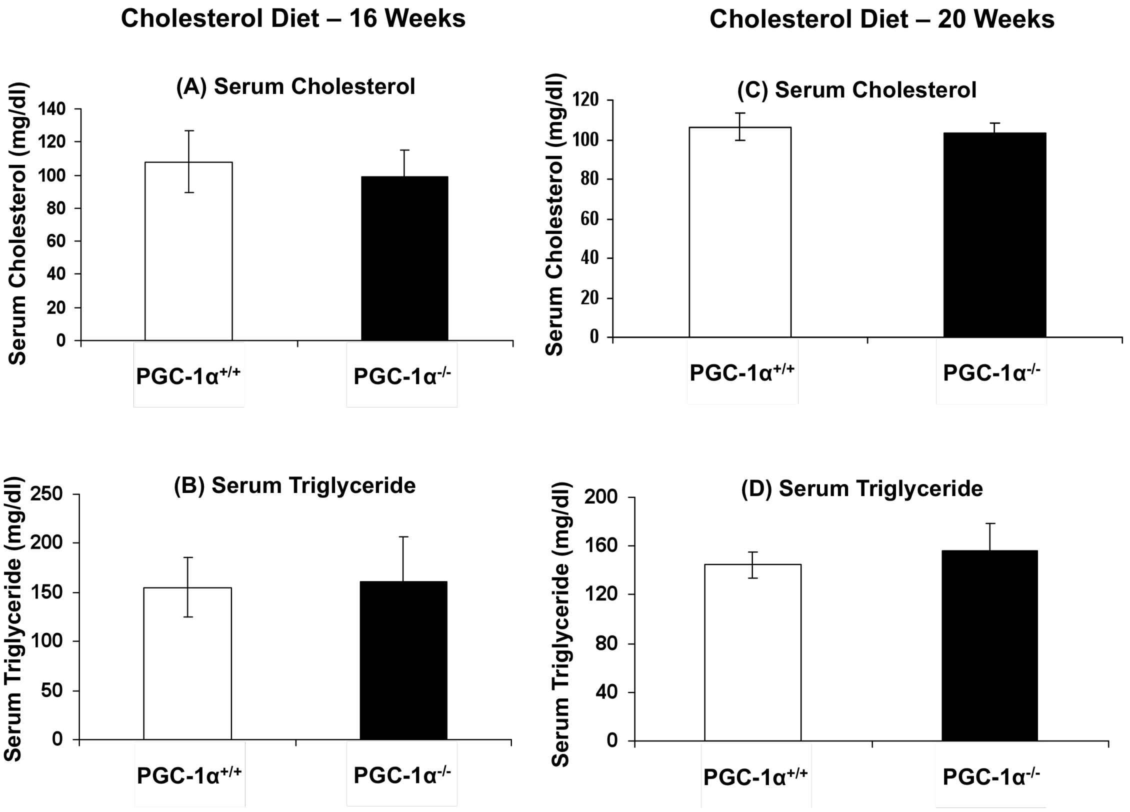

Supplementary Figure 9A. Serum cholesterol and triglyceride levels in PGC-1α$^{+/+}$ and PGC-1α$^{-/-}$ mice on a high cholesterol diet

Serum samples from PGC-1α$^{+/+}$ and PGC-1α$^{-/-}$ mice were analyzed for total cholesterol and triglyceride levels following either 16 or 20 week high cholesterol diet. Both genotypes showed similar concentrations of (A) serum cholesterol and (B) serum triglycerides after 16 weeks on diet. The results were similar in the 20 week fed groups with no difference in (C) serum cholesterol or (D) serum triglycerides.

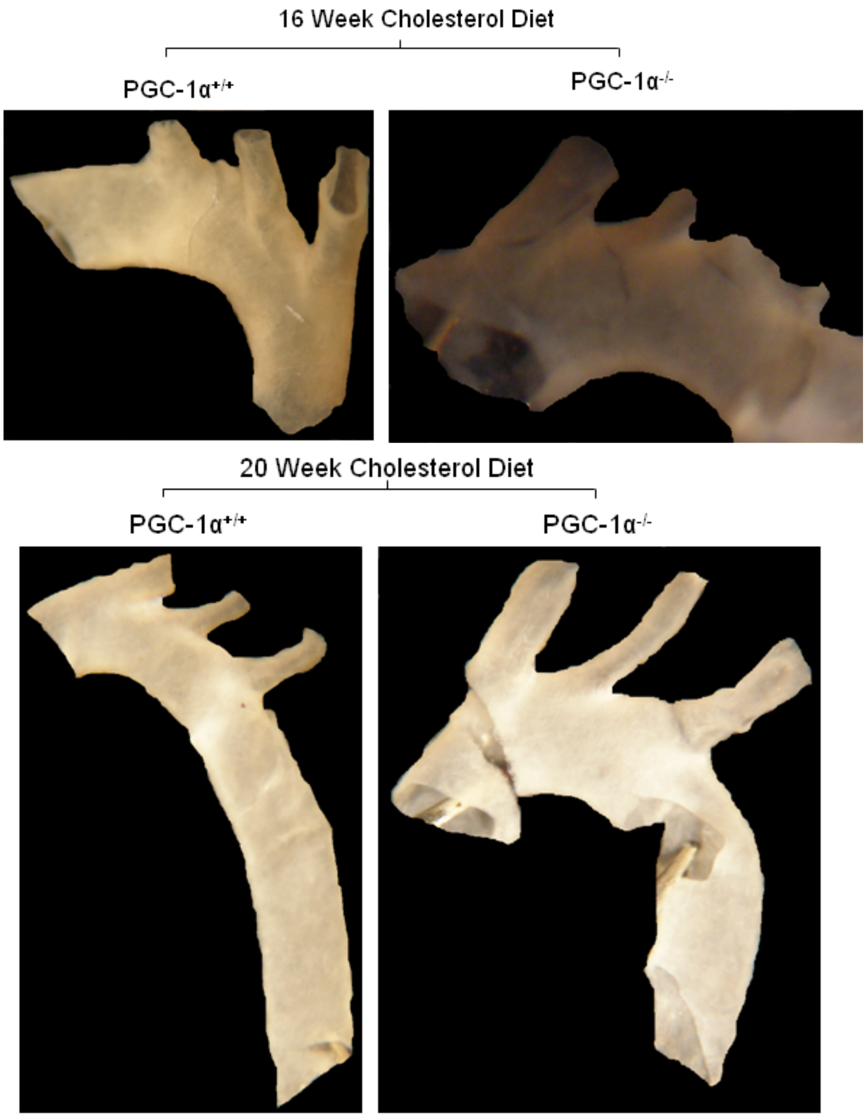

Supplementary Figure 9B. No evidence of atherosclerotic lesion development in the isolated aorta of PGC-1α$^{+/+}$ or PGC-1α$^{-/-}$ mice on a high cholesterol diet

The aortic root and arch was dissected from each mouse and *en face* analysis coupled with Oil Red O staining carried out to determine if lipid deposition had occurred. (A) Isolated aorta from 16 week cholesterol fed PGC-1α mice showed no signs of lipid deposition or plaque formation in either genotype. (B) Aorta from 20 week cholesterol fed PGC-1α also showed no signs of lipid deposition or plaque formation

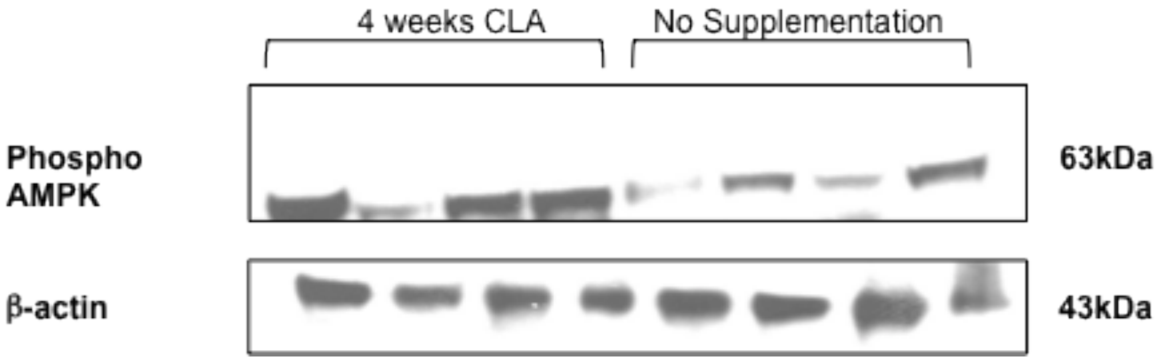

Supplementary Figure 10. CLA increases AMPK phoshorylation *in vivo*.

Western blot analysis of AMPK phosphorylation in murine aorta from apoE$^{-/-}$ mice that received a 1% cholesterol chow for 12 weeks (n=4) and those that received the same diet for 12 weeks where the final four weeks were supplemented with 1%CLA (n=4). β-actin was used as a loading control for Western blot analysis.

Supplementary Video Files 1 and 2

The following video files (Quicktime movies) demonstrate a 3-dimensional co-localization of PGC-1α with the macrophage marker F4/80 (murine aorta, video 1) or CD68 (human carotid artery, video 2). Tissue sections were imaged in an optimized 3D z-stack using confocal microscopy. Co-localization analysis using IMARIS software package generates an additional channel (grey), representing the co-localized voxels within the macrophage. Animated 3D renders were exported from IMARIS and assembled with Adobe After Effects CS4.

Supplementary Video 1. Murine aorta PGC-1α co-localization to macrophage cells. Murine aorta section immunofluorescently stained for macrophages with rat-anti-mouse-F4/80, followed by secondary anti-rat-IgG-Alexa-647 (red channel). Rabbit-anti-mouse-PGC-1α staining, followed by secondary anti-rabbit-IgG-Alexa-546 (green channel). Co-localization of $F4/80^+$ macrophages and PGC-1α are rendered in grey. DAPI stained nuclei are isosurfaced in blue.

Supplementary Video 2. Human atherosclerotic plaque PGC-1α co-localization to macrophage cells. Human carotid artery section immunofluorescently stained for macrophages with monoclonal mouse-anti-human-CD68, followed by secondary Alexa-594-goat-anti-mouse-IgG-$F(ab')_2$ fragment. Polyclonal rabbit-anti-human-PGC-1α staining, followed by secondary Alexa-647 goat-anti-rabbit-IgG. Co-localization of $CD68^+$ macrophages and PGC-1α are rendered in grey. DAPI stained nuclei are isosurfaced in blue.

Supplementary Video 3. oxLDL flux in PGC-1α– GFP transfected RAW macrophages. Time lapse imaging of GFP-PGC-1α transfected macrophages (green) and non transfected macrophages treated with oxLDL for four hours. In non-transfected cells oxLDL uptake and

accumulation is visible and the cells increase in size. In the GFP positive cells there is no oxLDL accumulation in the cells and the cell maintain their phenotype.

Supplementary Methods

All reagents used in this study were of the highest purity and unless otherwise stated, were obtained from Sigma-Aldrich (Dorset, UK).

Immunohistochemical and Western Blot Analysis of Murine Aorta and Human Atherosclerotic Plaque

For murine aortic tissue and human atherosclerotic plaque, 30µg of total protein was separated by SDS-PAGE under reducing conditions, followed by electrophoretic transfer of the proteins to nitrocellulose membrane. Antibodies used were polyclonal rabbit Anti-PGC1α (1:500, Calbiochem) for human and murine samples, polyclonal rabbit Anti-UCP-1 (1:1000, Calbiochem), monoclonal mouse CYP7B1 (1:500, Abnova), monoclonal mouse β-actin (1:500, Santa Cruz, Germany) for murine samples. For immunohistochemsitry analysis 5µm serial paraffin-embedded aortic sections were incubated with PGC-1α (1:1000), or the macrophage marker F4/80 (1:50) (Abcam Cambridge, MA) overnight at 4ºC.

Quantification of PGC-1α expression in human atherosclerotic plaque

The Aperio Software Analysis System *Nuclear Analysis* algorithm was applied to quantify the level of PGC-1α expression per cell and then the total of PGC-1α positive cells per tissue section. Nuclear analysis was then carried out on stained sections to establish the target protein expression firstly, as "expression per cell", and then as "a total per tissue section. This algorithm evaluated the intensity of DAB staining (protein staining) in each Haematoxylin (nuclei) stained cell and specifically and unequivocally assigned a mark-up image composed of different colours, corresponding with different levels of protein expression, which was counted and quantified. The algorithm used is outlined in the figure below.

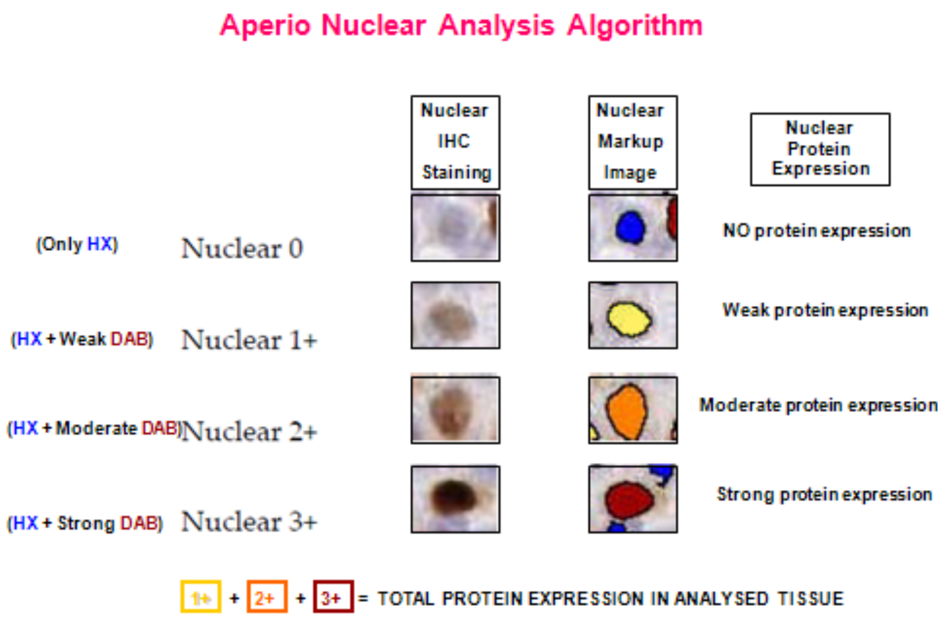

Supplementary Figure 11. Algorithm used in quantification of PGC-1α expression. The left panel demonstrates the intensity of the immunostaining. The right panel shows the corresponding mark-up image relative to the intensity of the staining and protein expression.

Cell Culture

The murine macrophage cell line RAW 264.7 was purchased from the LGC Promochem (Middlesex, UK) and cultured in Dulbeccos Modified Eagles Media (modified DMEM) supplemented with 10% fetal bovine serum and antibiotics (100 U/mL penicillin, 100 μg/mL streptomycin). Cells were grown in a humidified atmosphere of 5% CO_2/95% air at 37°C and sub-cultured every two days by gentle scraping. RAW 264.7 macrophages were treated for 24hrs with 25μM c9,t11-CLA, t10,c12-CLA, CLA blend (80:20 c-9t,11-CLA: and t10,c12-CLA), oleic acid (OA) or DMSO in 1000μL of modified DMEM containing 1% serum. Post treatment cells were exposed to 50μg/ml oxidised LDL (ox-LDL) (Intracel, DC, USA), either non-labelled ox-LDL RP-047, or Dil labelled RP-177 (Dil ox-LDL) for 4 hrs.

RNA isolation and Real Time PCR

Following CLA treatments and incubation with ox-LDL Raw 264.7, RNA was extracted using the RNeasy mini-kit (Qiagen). Reverse transcription was carried out using reverse transcriptase (Promega) according to the manufacturers instructions. Quantification of PGC-1α, UCP-1, PPARγ and Cyp7b1 gene expression by Real-Time PCR was performed on an optical-grade 384-well plate platform using a fluorescence-based, real-time detection method (ABI Prism 7700 Sequence Detection System, Applied Biosystems). RNA was extracted from aortic tissue of $apoE^{-/-}$ mice by homogenising tissue samples under liquid nitrogen. RNA was isolated using Trizol (Invitrogen, Bio-Sciences, Dublin) and further purified using RNeasy kit (Qiagen, UK). Protein was extracted using Tissue Protein Extraction Reagent (T-PER) (Fisher Scientific, Dublin). mRNA expression of PGC-1α, CPT-1, PPARγ and Cyp7b1 was performed as above.

Primer sequences for real time PCR analysis and PGC-1a Genotyping

SYBR Green Primers (MOUSE)

1. UCP1 FWD: CCTGCCTCTCTCGGAAACAA

REV: TGTAGGCTGCCCAATGAACA

2. Cyp7b1 FWD: GCCGATTATCAGCGAAAGCC

REV: GTGATTTTTAGCAGTTGGGAC

3. SRA1 FWD: GGGCCATTTTTAGTGCTGTGA

REV: TCCTGGTGCTCCTGGGTTT

PGC-1α Genotyping Primers (MOUSE):

HET FWD: CCAGTTTCTTCATTGGTGTG

HET REV: ACCTGTCTTTGCCTATGATTC

KO FWD: TCCAGTAGGCAGAGATTTATGAC

KO REV: CCAACTGTCTATAATTCCAGTTC

Flow cytometry

Cells were resuspended in 1ml PBS and Dil ox-LDL uptake was assessed by flow cytometry (Coulter EPICS XL-MCL). Forward and side scatter gates were established to exclude non-viable cells and cell debris from the analysis. Fluorescence signals from the accumulated Dil associated with the cells were detected at 555–600 nm by a photomultiplier and then converted to digital format and processed. The mean fluorescence intensity of 1.5-3 x 10^4 cells were analysed in each sample. Auto-fluorescence signals generated by unlabelled cells were used as negative controls in each experiment.

Cholesterol Uptake Assay

RAW 264.7 macrophages were plated at a density of 1.5 x 10^5 cells/well in a 12 well plate and incubated overnight. Cells were subsequently treated for 24hrs with 25µM c9, t11-CLA, t10, c12-CLA, CLA blend (80:20 c-9t, 11-CLA: and t10, c12-CLA), oleic acid (OA), linoleic acid (LA) (Cayman Chemicals, MI, USA) or DMSO in 1000µL of modified DMEM. Cells were washed twice prior to addition of DMEM containing 50µg/ml acLDL (Invitrogen) and 1uCi/ml triatiated cholesterol (Perkin Elmer). Cells were loaded for 4 hrs. Isopropanol (Sigma-Aldrich) was used to extract lipids, which were dissolved in tolulene (Sigma-Aldrich). Scintillation counts were determined using TriCarb 2900Tr Liquid Scintillation (Packard).

Cholesterol efflux Assay

To cholesterol-label macrophages, media containing 1% FBS, [^{3}H] cholesterol (1µCi/ml) and acLDL (25µg protein/ml) was added to the cells for 48 hrs. RAW 264.7 macrophages were incubated in media containing 0.2% BSA for 24 hrs to allow cellular cholesterol radiolabel to equilibrate. During the equilibration period cells were treated with 25µM CLA isomers or CLA blend, oleic acid, or DMSO. Time zero cells were harvested to determine total counts/min (cpm), in the cells before adding the extracellular cholesterol acceptors.

Media containing HDL (20µg/ml) or apoA-I (25µg/ml), 2% serum, or media alone was added for up to 4 h. [^{3}H]-Sterols in the media were compared to total [^{3}H] at time zero to determine the percent release of [^{3}H] cholesterol.

Plasma cholesterol, triglyceride and lipoprotein analysis

Mice were fasted for 4 hours, blood was collected, followed by centrifugation to isolate plasma. Total plasma cholesterol and triglycerides were measured using Cholesterol CHOD-PAP and Triglycerides GPO-PAP kits respectively (Roche, Mannheim, Germany). For lipoprotein analysis, lipoproteins were separated by fast protein liquid chromatography using an AKTA apparatus (column:SuperoseTH 6PC3.2/30). Cholesterol and triglycerides were measured in the collected fractions using the previously described kits. Measurement of lipoprotein profiles was conducted by TNO Biosciences, Leiden, The Netherlands.
